# Accretion of volatile elements on Earth without the need of a late veneer

**DOI:** 10.1126/sciadv.ady8018

**Published:** 2026-02-25

**Authors:** Lucas Calvo, Julien Siebert, Dongyang Huang, Ingrid Blanchard, Edith Kubik, Valentina Bonino, Anja Schreiber, Guillaume Avice, Jabrane Labidi

**Affiliations:** ^1^ Institut de Physique du Globe de Paris (IPGP), Université Paris Cité, Paris, France.; ^2^ SKLab-DeepMinE, MOEKLab-OBCE, School of Earth and Space Sciences, Peking University, Beijing 100871, China.; ^3^ Institut de Minéralogie, de Physique des Matériaux, et de Cosmochimie (IMPMC), Sorbonne Université, CNRS, Paris, France.; ^4^ Bayerisches Geoinstitut, Universität Bayreuth, Bayreuth, Germany.; ^5^ ESRF, The European Synchrotron, 71 Avenue des Martyrs, CS 40220, 38043 Grenoble Cedex 9, France.; ^6^ GFZ Helmholtz Centre for Geosciences, Potsdam, Germany.

## Abstract

Volatile elements are essential for life development and planetary evolution. However, the timing of their delivery to terrestrial planets remains unclear. Sulfur, selenium, and tellurium are volatiles, but also siderophile elements. Their abundances in Earth’s mantle can be used to determine whether volatile elements were delivered to Earth during or after the segregation of the core. Here, we experimentally measured their partition coefficients between core-forming metal and mantle silicate under pressure, temperature, and oxygen fugacity conditions relevant to a deep magma ocean. Our results show that these elements exhibit similar partitioning behaviors, indicating that core-mantle equilibrium preserves their chondritic relative abundances. If a volatile-rich late veneer has been delivered to Earth after core segregation, it must have been limited in mass, making up a maximum of 0.1% Earth’s mass. This suggests that volatile elements, including water, were accreted continuously during Earth’s growth rather than being delivered predominantly by a late veneer of volatile-rich material such as carbonaceous chondrites.

## Teaser

S, Se, and Te siderophile behavior in metal-silicate equilibration experiments is used to explain Earth’s accretion history.

## INTRODUCTION

Volatile elements—as defined by their low temperature of condensation (*1*) such as hydrogen (H), carbon (C), nitrogen (N), and sulfur (S)—are fundamental to Earth’s habitability and played an essential role in shaping its geodynamic processes. The timing and mechanism of delivery of volatile elements, as well as the amount delivered, are key to understanding the formation of Earth’s hydrosphere, the onset of plate tectonics, the geodynamic differences between Earth, Mars, and Venus, and the evolution of deep planetary reservoirs.

Sulfur, selenium (Se), and tellurium (Te) are siderophile and chalcophile, meaning that they have an affinity for iron (Fe) to form sulfides, and tend to partition into the core during Earth’s differentiation. They also share similar volatile behaviors, with relatively low condensation temperatures around 700 K under nebular conditions (*1*). Being moderately siderophile and volatile elements (MSVEs) makes S, Se, and Te ideal for investigating whether volatile elements were present during Earth’s primary accretion stages, when core formation was still occurring, or whether they were introduced later through late accretion, also known as the “late veneer” process [e.g., (*2*, *3*)]. The bulk silicate Earth (BSE) contains 217 ± 38 parts per million (ppm) S, 77 ± 38 parts per billion (ppb) Se, and 10 ± 5 ppb Te (*4*–*7*), and these elements are similarly depleted relative to chondrites—in chondritic-depletion—in part due to sequestration into the core (*8*) (Supplementary Text). Experimental studies up to 20 GPa and 2500 K predict that these elements become increasingly siderophile at higher pressures (*9*). Earth’s core formed at pressures between 50 and 60 GPa and temperatures between 3500 and 4000 K [e.g., (*10*–*15*)]. Extrapolations to these conditions predict that Te is far more siderophile than Se, which, in turn, is more siderophile than S, with differences in partitioning spanning several orders of magnitude (*9*). This discrepancy would indicate that the similar depletions of S, Se, and Te observed in the BSE cannot be explained only by metal-silicate equilibration during core formation. Instead, their substantial sequestration into Earth’s core followed by the addition of a late chondritic component, the late veneer, would account for their absolute and relative abundances (*9*, *16*). Although this hypothesis was originally introduced to explain the chondritic relative and elevated absolute abundance of highly siderophile elements (HSEs) in the BSE (*2*, *17*–*19*), the late accretion of volatile-rich carbonaceous chondrites (CCs) has also been proposed to explain the abundances of S, Se, and Te (*9*, *16*, *20*) and the volatile element budget in general of BSE (*16*, *21*).

In this study, we present experiments reproducing the simultaneous metal-silicate partitioning of S, Se, and Te under extreme pressure and temperature conditions relevant to core equilibration in a deep magma ocean. Our experiments were conducted at oxygen fugacity (*f*O_2_) directly relevant to core-mantle equilibration (~∆IW-2, referring to the iron-wüstite buffer), providing experimental constraints on the maximum S content in the core, as well as silicon (Si) and oxygen (O) abundances, yielding results consistent with recent estimates of core composition. The results presented here support the hypothesis that S, Se, and Te and, by extension, other volatiles were fully accreted during Earth’s primary accretion phases, negating the necessity for a late veneer contribution.

## RESULTS

**Fig. 1.**
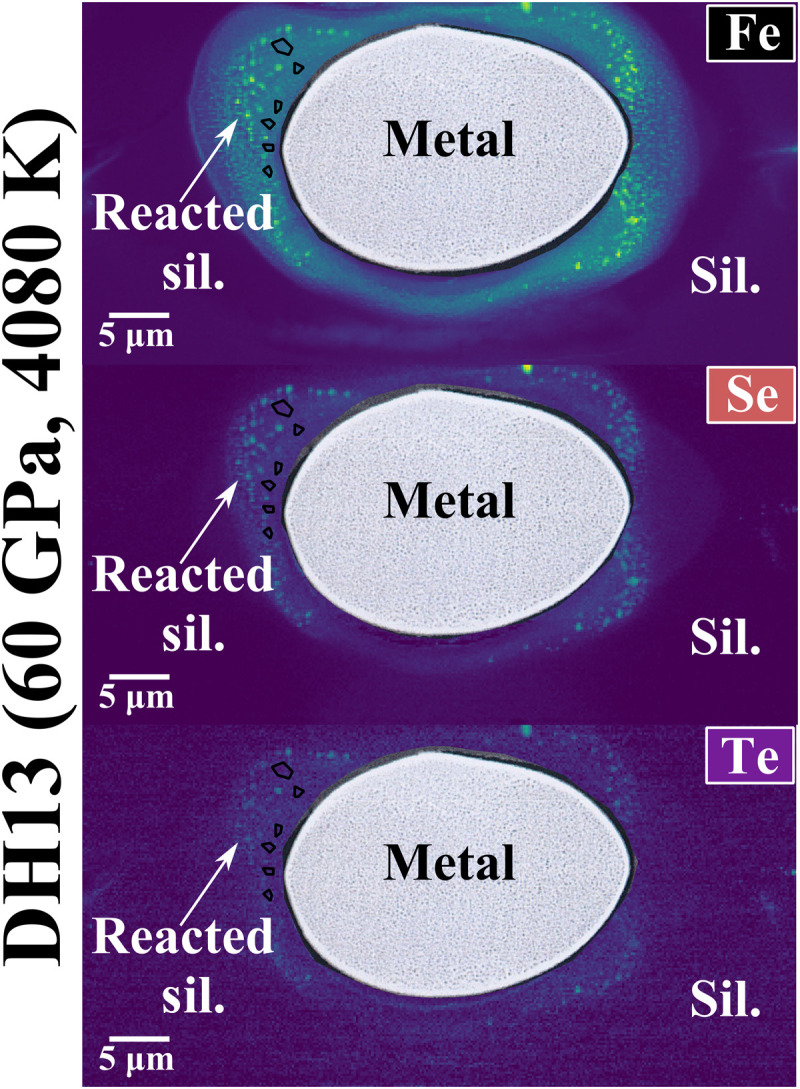
Backscattered electron images and overimposed nanoresolved XRF maps for Fe, Se, and Te obtained from sample DH13. Lower to high concentrations are represented in color scale from blue to green, respectively. Se and Te concentrations were obtained from the regions of interest marked by dark-edged polygons, avoiding the presence of big metal inclusions (Supplementary Text).

Six experiments using the laser-heated diamond anvil cell (LH-DAC) technique were conducted at pressures of 43 to 90 GPa and temperatures of 3610 to 4710 K (see Materials and Methods and table S1), conditions relevant to core-mantle equilibration in a deep magma ocean, which can explain the depletions of siderophile elements such as nickel (Ni), cobalt (Co), tungsten (W), vanadium (V), and chromium (Cr) (*10*–*15*). For each experiment, focused ion beam (FIB) sections of the run products show a metal liquid phase that coalesced in a single metal blob, in the center of the sample, surrounded first by a silicate melt and second by an unreacted portion of pyrolite (Fig. 1 and fig. S1; see Materials and Methods). Major elements and S concentrations in both phases were measured using electron probe microanalysis (EPMA). The precise quantification and distribution of Se and Te trace elements in the silicate phase was determined by nanoresolved x-ray fluorescence (XRF) mapping, collected at the ID16B nanoanalysis beamline of the European Synchrotron Research Facility (ESRF) (see Materials and Methods and table S1). This characterization technique provides both parts-per-million detection limit and high spatial resolution. Figure 1 presents quantitative XRF maps of the silicate melt, revealing trace abundances of Se and Te and Fe enrichment compared to the unreacted silicate, which is attributed to the incompatible behavior of Fe under the experimental conditions (*13*, *22*, *23*). The presence of metal inclusions in the silicate phase of these experiments does not substantially modify the silicate composition, and they presumably originate from exsolution during quench (Supplementary Text).

**Fig. 2.**
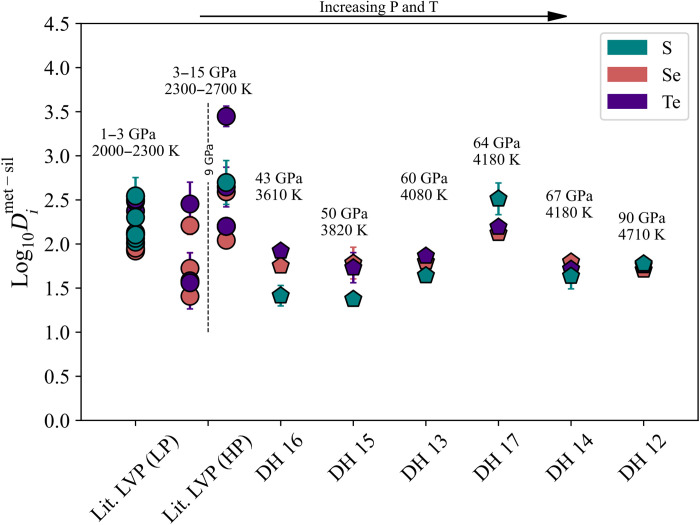
Partition coefficients measured for S, Se, and Te (in logarithmic units) for different metal-silicate experiments. Values from an experimental series in large-volume press experiments (LVP), separated arbitrarily at 9 GPa into lower (LP) and higher pressures (HP) [in circles; (*9*)], and our experiments (in pentagons, DH16 to DH12) show different metal-silicate partitioning behaviors. As equilibration temperature and pressure increase S, Se, and Te, partition coefficients converge to similar, less siderophile values.

We determined the partition coefficient of each element between metal and silicate phases through their molar ratio ($D_{i}^{\text{met}-\text{sil}}=X_{i}^{\text{met}}/X_{i}^{\text{sil}}$). These coefficients, suggested to be dependent on P, T, and *f*O_2_ (*9*, *24*), show less variation than predicted from low P-T conditions (*9*), with a convergence of log $D_{S,\text{Se},\text{Te}}^{\text{met}-\text{sil}}$ toward the highest P-T conditions explored here, and become indistinguishable from each other above 50 GPa (Fig. 2 and table S1). In addition, two experiments also present *f*O_2_ conditions similar to the redox state of the last final core-mantle equilibration process (DH15 and DH17, 50 to 64 GPa, 3820 to 4180 K, ∆IW = −1.8, −2). The liquid metals contain 74.09 ± 1.51% Fe, 3.65 ± 0.58% Si, and 4.00 ± 0.52% O, in weight % (wt %) (table S1), similar to the light elements abundance in Earth’s core predicted by mineral physics and geochemistry constraints (*4*, *5*, *25*). Such P-T-*f*O_2_ conditions directly relevant to core formation have been hardly achieved simultaneously in LH-DAC experiments, leaving them as unique observations for the budget of light elements in the core. The S, Se, and Te partition coefficients in these experiments unequivocally converge to the estimated core-mantle ratio of these elements on Earth with $\text{log}\;D_{i}^{\text{met}-\text{sil}}$ of 1.92 ± 0.10, 1.93 ± 0.09, and 1.94 ± 0.09, for S, Se, and Te, respectively. By themselves, these results suggest that a maximum of 2 wt % S could be stored in the core to account for the observed S composition of the BSE (*4*, *5*, *8*, *25*). This also means that, if the material that formed the Earth is mostly chondritic (*26*–*28*), S, Se, and Te chondritic relative abundances would be preserved as they would partition in equal relative amounts into the core.

We compiled our results and those of lower pressure and temperature experiments (Supplementary Text and tables S2 and S3) to parametrize the dependency of S, Se, and Te partition coefficients as a function of T, P, and FeO content in the mantle, and sulfide capacity following thermodynamical constraints (Supplementary Text). Using a multilinear parametrization approach (Supplementary Text), we obtain a stronger negative effect of temperature on the partitioning of S, Se, and Te and a notably weaker positive effect of pressure compared to previous studies (table S4) (*9*). Including a fourth coefficient to account for the O molar fraction in the metal yields statistically insignificant values (*P* < 0.05; Supplementary Text and table S4). Therefore, we distinguish no significant effect of the light element composition of the metal alloy, particularly O, on the partitioning of these elements. Using the calculated parametrization, we can predict how S, Se, and Te were distributed between core and the silicate Earth during its accretion and differentiation (Supplementary Text).

## DISCUSSION

**Fig. 3.**
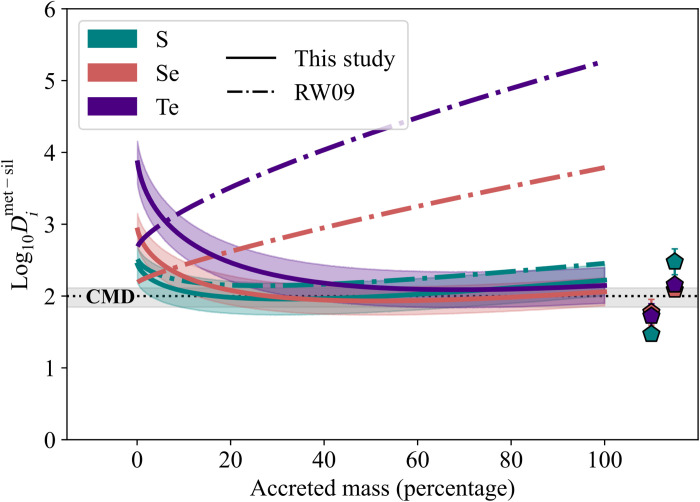
Partition coefficients calculated for S, Se, and Te (in logarithmic units) as a function of accreted mass to Earth (in percentage). Calculations were performed under constant oxygen fugacity conditions (log *f*O_2_ = −2.3 ∆IW, *X*^mantle^_FeO_ = 0.057). Evolution of these elements in this study (continuous lines) converges toward the estimated Earth’s current core-mantle distribution (CMD) (gray band; $D_{S,\text{Se},\text{Te}}^{\text{met}-\text{sil}}∼100$), whereas studies only considering low P-T experiments (RW09, dashed lines) diverge and move away from the CMD (*9*). Small differences between the RW09 and Rose-Weston *et al.* (*9*) models are due to calculated equilibration temperatures following more recent studies (*61*, *62*). DH15 and DH17 (left and right, respectively) are plotted for comparison with the model results (see main text). Our results indicate that chondritic relative abundances can be obtained solely by metal-silicate partitioning of these elements. Errors on the model rise from a Monte Carlo approach (Supplementary Text).

We have modeled core formation as a continuous process during Earth’s accretion, considering increasing P-T conditions of core-mantle equilibration as the magma ocean deepens [e.g., (*10*–*15*) and (*29*–*31*)]. We find that the partition coefficients of S, Se, and Te between metal and silicate decrease with increasing P-T of equilibration, meaning that, during planetary growth, the siderophility of S, Se, and Te decreases. At pressures above 50 GPa, the metal-silicate distribution of the three elements is indistinguishable from each other and from the required Earth’s core-mantle distribution, reaching values of $\text{log}\;D_{S}^{\text{met}-\text{sil}}=2.22\pm 0.22,\text{log}\;D_{\text{Se}}^{\text{met}-\text{sil}}=2.06\pm 0.20$, and $\text{log}\;D_{\text{Te}}^{\text{met}-\text{sil}}=2.15\pm 0.25$ (Fig. 3). These results are different from previous experimental data (*9*). Using the parametrization based on low P-T experiments, partition coefficients reach values of $\text{log}\;D_{S}^{\text{met}-\text{sil}}=2.46\pm 0.22,\text{log}\;D_{\text{Se}}^{\text{met}-\text{sil}}=3.79\pm 0.20,\text{and}\;\text{log}\;D_{\text{Te}}^{\text{met}-\text{sil}}=5.27\pm 0.25$ (Fig. 3). The uncertainties given are those arising from our parametrization as no uncertainty was reported in the original study. This would mean that BSE abundances of S, Se, and Te after core-mantle equilibria would be strongly nonchondritic, with absolute abundances below observations for the BSE (*4*–*7*). This would naturally require the late addition of a chondritic late veneer. Our results, conducted under direct conditions of core formation, challenge this hypothesis.

We simulated the BSE S, Se, and Te contents in two endmember scenarios: homogeneous volatile delivery throughout the entire accretion process [e.g., (*32*)] versus volatile delivery constrained to the final 20% of accretion, reflecting heterogeneous composition of material delivered to Earth [e.g., (*33*–*37*)] (see the Supplementary Text for details and references therein). Both scenarios are constructed to generate bulk Earth concentrations of S, Se, and Te of 6350 ppm S, 2700 ppb Se, and 300 ppb Te, respectively, estimates that rise from cosmochemical constraints (*5*, *8*). In the case of homogeneous accretion, we take Earth’s building blocks to have volatile contents equal to that of bulk Earth throughout the whole accretion sequence. For the heterogeneous accretion scenario, we take volatile-free bodies until 80% of Earth’s mass is achieved, followed by the accretion of volatile-rich bodies akin to chondrites with abundances of ~32,000 ppm S, ~13,600 ppb Se, and ~1500 ppb Te (Supplementary Text). We evaluated the effect of four different *f*O_2_ paths evolving toward a final value of ∆IW-2.3, by testing initial magma ocean compositions either being oxidized (∆IW-1.5 and ∆IW-1.9), reduced (∆IW-4.5), or constant (∆IW-2.3) (paths A to D, Supplementary Text and figs. S2 and S3).

**Fig. 4.**
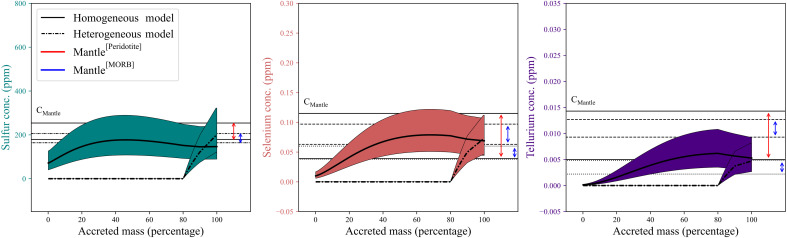
S, Se, and Te concentrations in the BSE as a function of accreted mass to Earth. This was calculated by stepwise mass balance under constant oxygen fugacity conditions ($\text{log}fO_{2}\;\mathrm{\Delta IW}=-2.3$). Material ressembling Earth’s bulk S, Se, and Te abundance is added at each step, either in a homogenous or heterogeneous manner [e.g., (*32*–*37*)]. Several estimates have been proposed for the composition of the mantle (*4*, *6*, *42*). The red arrows denote the upper and lower bounds of BSE concentrations inferred from peridotite samples (*4*, *6*). In addition, two alternative estimates, derived from analyses of mid-ocean ridge basalt (MORB) samples, are indicated with blue arrows (*42*). No scenario is strictly dependent on a late veneer contribution to match them.

We find that in all the simulations for all modeled *f*O_2_ paths using our set of partition coefficients, the final abundances of S, Se, and Te match the observed BSE abundances within uncertainty (Fig. 4 and figs. S4 and S5). This is true for homogeneous volatile accretion, where concentrations reach $146_{-57}^{+93}$ ppm S, $69_{-25}^{+38}$ ppb Se, and $5_{-2}^{+4}$ ppb Te. This is also true for heterogeneous volatile accretion, reaching final abundances of $197_{-77}^{+125}$ ppm S, $72_{-26}^{+40}$ ppb Se, and $5_{-2}^{+4}$ ppb Te (Fig. 4), slightly higher than those observed for homogeneous delivery due to the enhanced partition coefficient of S, Se, and Te early during Earth’s accretion (Fig. 3). Furthermore, we evaluated the effect of metal-silicate segregation combined with evaporative loss of these elements (*38*, *39*). Regardless of the complexity of such a scenario, we tested their assumptions and found that Earth’s S, Se, and Te budget can still be matched by metal-silicate equilibria (Supplementary Text and fig. S6) (*38*, *39*). In addition, neither sulfide segregation (*40*) nor Fe disproportionation (*41*) substantially influence the budget of these MSVEs in the BSE (Supplementary Text). Therefore, we conclude that core-mantle segregation is the main event that can explain Earth’s current distribution of S, Se, and Te.

The late addition, after core-mantle segregation, of up to 0.5% Earth’s mass material of CC-like material, being either Mighei (CM) or Ivuna (CI) type, has been used to account for the abundance of HSEs (*3*), S, Se, and Te in the BSE with the concomitant delivery of other volatiles at the Earth’s surface, such as C and water (*16*). The delivery of 0.5 wt % CI-type material (table S5) to Earth’s mantle after our homogeneous model estimate of high-pressure metal-silicate fractionation would produce a BSE with $514_{-57}^{+93}$ ppm S, $203_{-25}^{+38}$ ppb Se, and $22_{-2}^{+4}$ ppb Te. The same amount of CM-type material (table S5) would yield a BSE with $386_{-57}^{+93}$ ppm S, $167_{-25}^{+38}$ ppb Se, and $16_{-2}^{+4}$ ppb Te. Both cases would produce chondritic relative abundances, yet inevitably leaving too elevated absolute abundances to be reconciled with BSE estimates. Unless complex models of S, Se, and Te depletions during planetary evolution are invoked to match the data, the addition of 0.5% Earth mass of CC appears challenging to reconcile with observations, considering our partitioning data. In contrast, the addition of 0.1 wt % mass of CI-like material would produce final concentrations of $220_{-57}^{+93}$ ppm S, $96_{-25}^{+38}$ ppb Se, and $8_{-2}^{+4}$ ppb Te. Although it would not satisfy HSE abundances in the mantle, this would fit S, Se, and Te estimate for BSE concentrations (*4*–*7*, *42*).

Considering the relatively large uncertainties reported here, naturally associated with geochemical mass balances in evaluating late veneer scenarios, we take an additional step to determine the statistical significance of all possible scenarios between 0.1 and 0.5 wt % of Earth’s mass addition of chondritic-like materials to BSE abundance after core formation. For the statistical analyses, we take peridotite-derived compositions as a proxy for the mantle (*4*, *6*). By performing *t* tests, we evaluated whether this late addition would be statistically different from BSE estimated composition. In addition, we evaluated the consistency between both of them by Bayesian inference tests, which tell the degree of overlap between probability density functions. The combination of both statistical methods, with the condition that tested scenarios should agree with BSE estimates for all three elements, S, Se, and Te, on the statistical significance, gives constraints on the amount and composition allowed for the late veneer event. Successful cases were those showing a probability overlap higher or equal than 1σ and statistically significant similarity between means (probability of ≥0.68, *P* ≥ 0.05). We found that S and Te concentrations allow for a CI-like addition of up to 0.10 wt % or for a CM-like addition of up to 0.10 to 0.20 wt % of Earth’s mass. However, these constraints do not apply for Se, as concentrations in the BSE are statistically matched without any late veneer contribution (fig. S7 and table S6). A good compromise that matches S, Se, and Te BSE abundance in the models could be obtained by allowing up to a 0.07 wt % CI-like or 0.10 wt % CM-like material of Earth’s mass, accounting for a significant overlap probability despite statistically different means (highest overlap being > 0.68, *P* < 0.05; table S6). An alternative scenario involves the addition of volatile-depleted material such as ordinary chondrites (OCs), which have lower concentrations in S, Se, and Te than CCs but similar HSE concentrations (*43*). Considering the S, Se, and Te concentrations on OCs (table S5), a maximum of 0.25 wt % is allowed for matching their observed BSE concentration (table S6). After core-mantle segregation, this would produce a silicate Earth with $228_{-57}^{+93}$ ppm S, $100_{-25}^{+38}$ ppb Se, and $7_{-2}^{+4}$ ppb Te, consistent with BSE. Therefore, the mass balance exercise suggests that, at most, only 25% of total S, 28% of total Se, and 32% of total Te could come from the late veneer.

The interplay between S, Se, Te, and HSE indicates that any late addition to Earth must have been either volatile-poor or limited in mass. If the late veneer consisted of CI chondrites (*20*), our constraints impose an upper bound of ∼0.1 wt % of Earth’s mass. This conflicts with earlier proposals that 2 to 5 wt % of CI-like material was needed to explain Earth’s mantle inventory of highly volatile elements (*21*). Our upper limit is, however, consistent with the estimate of 0.15 ± 0.03 wt % from Varas-Reus *et al.* (*20*). Samples recently collected from the Ryugu asteroid show CI-like concentrations of S, Se, and Te but contain two to three times more water (*44*, *45*). Should the late veneer have been dominated by such Ryugu-like CI material, this could have delivered substantial water to Earth, yet its budget cannot be solely explained by a late veneer delivery. The addition of a more volatile-depleted, CM-like material comprising 0.20 wt % of Earth’s mass has been proposed to explain early Earth’s mantle isotopic ruthenium (Ru) anomalies (*46*). This should be taken, at most, as an upper limit given not only the results presented here but also that S and Se isotopic signature of BSE compared with CM (*20*, *42*, *47*, *48*) argue against a substantial chondritic late contribution. A late veneer comprising a maximum of 0.20 wt % of CI/CM-like material would not account for the HSE absolute abundances in BSE (*49*), as it leaves 6 to 11 times lower concentrations than observables for the silicate Earth. However, the effect of core-mantle equilibrium on HSE abundances, particularly for platinum (Pt) (*31*), may have been underestimated, and their partitioning need to be reevaluated under the direct P-T conditions of a deep magma ocean.

Volatile element–bearing materials are presumably Earth’s main building blocks [e.g., (*28*, *50*)]. Therefore, the partitioning data presented here indicate that Earth has set its volatile budget during the main stage of accretion and that its core contains a maximum of 2 wt % of S (fig. S4), if bulk Earth contains 6350 ppm S according to cosmochemical estimates [e.g., (*5*)]. The constraints on mass and composition of the late veneer material indicate that other volatile elements’ budgets, such as those of C, N, H, and noble gases, were also set during Earth’s main stage of accretion (*50*–*58*) and, by extension, other terrestrial bodies such as Mars (*59*). This implies that volatile-rich bodies were scattered into the inner Solar System during Earth’s main accretion stages, as suggested by recent dynamical models [e.g., (*60*)]. Consequently, volatile elements and habitability are no longer tied to the very late stages of Earth’s accretion.

## MATERIALS AND METHODS

### Starting material and experimental procedure

For the starting material, we synthesized a pyrolytic glass, to simulate primitive Earth’s mantle composition (*4*), from high-purity chemical reagents and carbonates (Al_2_O_3_, MgO, FeO, SiO_2_, and CaCO_3_). They were ground and mixed in ethanol using an agate mortar and decarbonated at 950°C, to create glass beads with an aerodynamic levitation furnace at the Institut de Physique du Globe de Paris (IPGP). We used an argon (Ar) flux on the furnace coupled with a 75-W CO_2_ laser (λ = 10.6 μm). The glassy starting material was polished into ∼20 μm and cut into small disks using a picosecond laser at the IPGP to place them between the diamonds on the DAC. The metal alloy used in the experiments was synthesized by piston cylinder press. A mixture of metal, oxide powders, and a natural MORB was molten at 2 GPa and 1400°C. The quenched run products consisted of a coalesced Fe–S–Se–Te alloy that was separated mechanically from the quenched silicate glass.

Using LH-DAC, we equilibrated both metal and silicate phases under conditions relevant to Earth’s core formation (43 to 90 GPa and 3610 to 4710 K) (table S1). The starting material was placed in a layered-like structure, with pyrolite glass disks at both ends, on the top and bottom of the metal alloy. We used preindented rhenium (Re) gaskets (40- to 50-μm thick) with flat culet diameters of 200 to 300 μm and then created the sample chamber by drilling a 100- to 150-μm diameter hole with a picosecond laser. We compressed the samples to target pressure and then laser-heated from both ends using a fiber laser (λ = 1070 nm, 200 W), with a focused beam size of ∼20 μm. Targeted temperature was increased beyond the pyrolytic liquidus (*61*, *62*). Few seconds are needed for attaining the metal-silicate equilibrium at such high pressure and temperatures (*13*, *63*). Laser power was ramped and held between 20 and 60 s at the final temperature before quenching by turning off the laser. We measured the heating temperature from both sides of the laser-focused spot by continuously fitting the Planck black-body function from the visible portion of the black-body radiation (500 to 750 nm). The estimated uncertainty is ±250 K. Pressure was determined by Raman spectroscopy of the diamonds before and after the experiments (*64*). We applied corrections for thermal pressure (∆*P* = 2.7 MPa/K) estimated from in situ studies with a similar experimental protocol (*13*).

### Sample recovery and textural analyses

We extracted sample and standard lamellae using a Ga^+^-FIB instrument equipped with a field-emission gun (FEG Zeiss Auriga 40) at the IPGP and a Helios G4 UC DualBeam system (Thermo Fisher Scientific/FEI) equipped with FEI’s Tomahawk FIB and an Elstar high-resolution field-emission scanning electron microscopy (SEM) column at Helmholtz Centre for Geosciences. They were removed from the gaskets and welded into transmission electron microscopy copper grids. The thin section dimensions are ∼30 μm by 15 μm by 3 μm. They were coated with a 10-nm-thick layer of graphite to ensure conductivity and were first characterized by SEM using backscattered electron imaging and energy-dispersive x-ray analyses. The run products show a liquid metal phase that reacted with a silicate melt, surrounded by unreacted silicate (Fig. 1 and fig. S1). The silicate phases seem homogeneous, despite the conspicuous presence of metal blobs, a feature observed in large-volume press [e.g., (*65*, *66*)] and LH-DAC experiments [e.g., (*14*, *24*, *67*)]. Their implications are discussed below in regard to their possible origins and consequences on the interpretations. Metals show small exsolutions (Fig. 1 and fig. S1) possibly formed upon quench, as observed in previous studies as well [e.g., (*31*, *68*)]. The low errors on the measured composition indicate homogeneous composition and, thus, equilibrium attainment (table S1).

### Electron probe and nanoresolved XRF compositional analyses

Major element compositions in both metal and silicate phases were acquired by wavelength-dispersive x-ray spectroscopy in EPMA on a Cameca SX-Five at Camparis. For these analyses, we used an acceleration voltage of 20 keV. The current beam was of 10 nA with peak counting times of 10 s for major elements [aluminum (Al), magnesium (Mg), calcium (Ca), Fe, and Si] in the silicate phases and for Fe in metals, whereas for minor and trace elements in both phases (e.g., Si, O, Mg, Al, and S in the metal), we used 40 nA and 30 s. We used 15 keV and 100 nA with long counting (60 s) on Se and Te. For most elements, the detection limit was 100 to 400 ppm. The standards used were diopside for Ca and Mg, orthoclase for Al and Si, hematite for Fe and O, BaSO_4_ for S, and metallic Se and Te. All elements were analyzed using the K_α1_ line, except Te, which was analyzed using the L_α1_. The spot size used was ∼1 μm, and when measuring the silicate, measurements were performed in areas other than that adjacent to the metal to avoid their influence. The thickness of the FIB section (3 to 4 μm) accounts for reliable analyses (*69*).

To avoid accounting for metallic inclusions and obtain optimized spatial resolutions for these types of samples, we acquired compositional nanoresolved XRF maps on the recently extremely brilliant source-upgraded beamline ID16B at the ESRF, which has been proven to account for high spatial resolution below 100 nm and detection limits in the parts-per-million level (*70*), already tested on LH-DAC samples (*71*). For such purposes, we used a pink beam mode and an excitation energy of 33.4 keV, with flux intensity at time *t* and time 0 of 1.54 × 10^12^ and 1.61 × 10^13^ for synchrotron session ES1306, and 1.18 × 10^12^ and 1.08 × 10^13^ ph/s for session ES1467, respectively. Two multielemental Si drift detectors (3 + 7 elements) were used for the acquisition. This configuration privileged Se and Te quantification, leaving S with a reduced x-ray absorption, not sufficient for its quantification. Therefore, S quantification was measured by EPMA. We conducted analyses on a FIB lamella of an internal standard for comparison with values obtained by nanoresolved XRF. This glassy standard (E595) was synthesized from a starting material of composition presented in table S7, modified from LRMS34 (*9*) in a piston cylinder experiment equilibrating metal and silicate phases at 2 GPa and 1500°C for 1 hour, with a run product composition described in table S8. We performed high-resolution mapping of areas at least 20 μm by 20 μm with a resolution higher than 100 nm and long exposure times (300 to 1000 ms), which accounted for detecting Se and Te at extremely low concentrations with the advantage of avoiding the presence of metal blobs in the silicate melt (see below). The compositions were extracted using the softwares PyMCA (*72*) and Silx view (*73*). We collected the composition from the spectra (fig. S8), verifying that elements such as Fe and manganese (Mn) showed similar abundances to those obtained by EPMA, although small differences may be expected due to the presence of the inclusions, which may be integrated into the 1-μm spot size of the microprobe analyses. Compositions between both methods are matched on Se and Te, as well as on major elements such as Fe and Mn.

## Supplementary Materials

### The PDF file includes:

Supplementary Text Figs. S1 to S14 Legends for tables S1 to S8 References
