# Supplementary material for: Accretion of volatile elements on Earth without the need of a late veneer
Source: Sci Adv. 2026 Feb 25;12(9):eady8018. doi: 10.1126/sciadv.ady8018 (PMC12935040; doi:10.1126/sciadv.ady8018)
Supplement: Supplementary file 1 — Supplementary Text Figs. S1 to S14 Legends for tables S1 to S8 References [file sciadv.ady8018_sm.pdf]

Science Advances
AAAS

Supplementary Materials for

Accretion of volatile elements on Earth without the need of a late veneer

Lucas Calvo *et al.*

Corresponding author: Lucas Calvo, calvo@ipgp.fr; Dongyang Huang, dhuang@pku.edu.cn

Sci. Adv. **12**, eady8018 (2026)
DOI: 10.1126/sciadv.ady8018

The PDF file includes:

Supplementary Text
Figs. S1 to S14
Legends for tables S1 to S8
References

Other Supplementary Material for this manuscript includes the following:

Tables S1 to S8

Supplementary text

The chondritic-depletion of S, Se, and Te

The bulk silicate Earth (BSE) contains 217±38 ppm S, 77±38 ppb Se, and 10±5 ppb Te (*4–7*). These abundances are based on the estimated S content of the BSE and assumed CI-chondrite-like ratios S/Se=2635 and Se/Te=8.92 ratios, which are supported by peridotite measurements that exhibit S/Se=2690±700 and Se/Te=7.9±1.6 (*ref. 16*). Notably, it is observed that S, Se, and Te are similarly depleted in the BSE relative to chondrites by a factor of ~250. As volatile elements, this could result from partial condensation or evaporative loss during or before planetary formation (*74–76*). Zinc (Zn) is an element of similar condensation temperature, and it is chalcophile and only mildly siderophile. The BSE is depleted in Zn by a factor of ~ 6 with respect to CI, meaning that the S, Se and Te BSE depletion by a factor of ~ 40 with respect to Zn (*8*) must be produced by the partitioning of these elements into Earth's core.

Metal inclusions in the reactive silicate and equilibrium attainment

The scanning electron images of our run products show the presence of metal inclusions in the quench silicate melt (fig. S1). These features have been reported in several experimental studies using different experimental apparatuses at varying conditions of pressure and temperature (*9, 36, 67, 77–80*). However, only in a few studies, they were put into discussion. As reported in Boujibar et al., (*65*), they could generate from A) brief heating time and thus, inefficient metal–silicate segregation, B) metal stripping due to strong silicate convection, C) metal exsolution from the silicate upon quenching (*77, 81–84*), or D) saturation at high pressure and temperature (*85*). The homogeneous distribution of metal inclusions (*14, 24, 65*), showing a different composition from the large metal blob (*65*) has been used as evidence for quench textures. Therefore, taking advantage of the analytical techniques used here, we observe their homogeneous distribution (Fig. S1), and no compositional difference when integrated in the silicate composition (Fig. S9). Throughout our experimental P–T range (DH16, DH13 and DH12), ΔSe^{IB-BF} (integrated blobs vs blob-free silicate compositions) decrease with increasing P–T from 19 to -3%, and ΔTe^{IB-BF} from 4 to -10%, adopting negative values at the highest P–T conditions. Consequently, we argue these are quench textures.

S, Se, and Te measured concentrations show no significative variation when performing compositional profiles, indicating equilibrium is achieved (Fig. S10). In order to be conservative, we report the composition of the silicate phases avoiding metal inclusions, even though they would not change substantially its composition.

In this study, S concentration in the silicate melt was obtained by EPMA since X-ray fluorescence nano-analysis was optimized for Se and Te elements excitation. As discussed above, the use of ~1 μm beam spot size with EPMA which integrates metal inclusions, does not affect the silicate composition. The relatability between the partition coefficient obtained here and those of previous studies with nano-SIMS (*24*) validates our results (Fig. S11).

Parametrization of element partitioning

We describe the distribution between metal and silicate of S, Se and Te as the partition coefficient ($D_i^{\mathrm{met-sil}}$), dependent on several thermodynamic variables, like pressure, temperature, composition and oxygen fugacity (*65, 67, 86*). Under reducing conditions, below the iron–wüstite (IW) buffer, sulfur dissolves into the silicate structure occupying oxygen atomic positions (*87, 88*) through the reaction R1:

(R1)
$$\mathrm{O^{2-}} + 0.5\ \mathrm{S_2} \leftrightarrow \mathrm{S^{2-}} + 0.5\ \mathrm{O_2}$$

If a large amount of oxygen atoms is in the anion form O^{2-}, the oxygen activity can be regarded as constant, and thus, we can consider the sulfide capacity (C_S, Eq. 1), which is the equilibrium constant of reaction R1 (*88*):

(Eq. 1)
$$C_S = \mathrm{X}_S^{\mathrm{sil}} \left(\frac{f\mathrm{O_2}}{f\mathrm{S_2}}\right)^{0.5}$$

Where $f\mathrm{O_2}$ and $f\mathrm{S_2}$ are the oxygen and sulfur fugacity, respectively. The sulfide capacity for silicate melts of different compositions has been empirically modelled from experimental studies at different oxygen and sulfur fugacity conditions and sulfur concentrations (Eq. 2) (*89*).

(Eq. 2) $\log C_S = -5.704 + 3.15 \pm 0.64\, X_{FeO}^{sil} + 2.65 \pm 0.56\, X_{CaO}^{sil} + 0.12 \pm 0.38\, X_{MgO}^{sil} + 0.77 \pm 0.91\, X_{TiO_2}^{sil} + 0.75 \pm 0.58\, (X_{Na_2O}^{sil} + X_{K_2O}^{sil})$

When we consider the equilibrium between FeS-rich metal and a silicate (Reaction R2):

(R2) $$FeO^{sil} + 0.5\, S_2 \leftrightarrow FeS^{met} + 0.5\, O_2$$

Consequently, considering Eq. 1, the Gibbs free energy reaction of R2 can be defined as (Eq. 3):

(Eq. 3) $$\frac{\Delta G^{\circ}\,(R2)}{2.303\, RT} = \log a_{FeS}^{met} - \log a_{FeO}^{sil} + \log C_s - \log X_S^{sil}$$

Where the activity of FeS is $a_{FeS}^{met} = \gamma_{FeS}^{met}\, X_{FeS}^{met}$. By taking the approximation of a constant activity coefficient γ_{FeS}^{met} as a function of pressure and temperature, we can replace it for a constant *a*: $a_{FeS}^{met} = a\ X_{FeS}^{met}$.

Consequently, we can calculate the distribution of S between metal and silicate as Eq. 4:

(Eq. 4) $$\log D_S^{met-sil} = \log\left(\frac{X_S^{met}}{X_S^{sil}}\right) = \log a_{FeO}^{sil} - \log C_S - \frac{\Delta G^{\circ}\,(R2)}{2.303\, RT}$$

Assuming a similar behavior for the three elements (S, Se and Te) due to their geochemical affinities, we can parameterize D from Eq. 4 by considering the effect of composition and oxygen fugacity from the first two terms, as well as the pressure and temperature dependency on entropy, enthalpy and reaction volume contributions on the third term (Eq. 5), with the consideration of a constant γ_{FeO}^{sil} in our range of pressure and temperature.

(Eq. 5) $$\log_{10} D_i^{met-sil} = a + \frac{b}{T} + c\, \frac{P}{T} + \log X_{FeO}^{sil} - \log C_s$$

We therefore compiled several studies and obtained *a*, *b* and *c* parameters (see below, Tables S2, S3, and S4). At the pressures and temperatures of our study, light elements like silicon and oxygen can be dissolved into the metallic phases (*13*). However, silicon is assumed to not

significantly influence the partitioning of these MSVE between metal and silicate. First, at pressures above 15 GPa, experiments show that (Fe,S) and (Fe,Si) alloys mix ideally, forming an Fe–S–Si system with near-ideal behavior (*90, 91*). This agrees with results at 20–25 GPa and 2273 K, which found no measurable effect of S on Si behavior in metal, even at S levels higher than in our experiments (*92*). Second, the Si contents in the metals of our experiments are relatively low (3.57–9.99 wt.% Si; Table S1). Within this range, we observe no resolvable effect of Si on the partitioning of S, Se, or Te under our P–T conditions. Finally, at lower P–T conditions (previous large-volume press experiments), S and Si are also not present together in high enough amounts to evaluate a significant effect on S partitioning. To further test this effect, we used interaction parameters in between S and Si from the Steelmaking Sourcebook (*93, 94*) which give an interaction parameter $\varepsilon_{\mathrm{Si}}^{\mathrm{S}}$= 8.9 at 1 bar—thus representing an upper-limit case. Calculations comparing two scenarios, one with high Si ($X_{\mathrm{Si\ metal}}$ = 0.15, similar to experiment DH12) and one with no Si, show negligible differences in the activity coefficient of FeS (γ_{FeS} = 0.29 with Si and γ_{FeS} = 0.16 without Si). Similarly, considering self-interaction of S ($\varepsilon_{\mathrm{S}}^{\mathrm{S}}$=-5.6) for up to 18 wt.% S only slightly decreases γ_{FeS} (from 0.29 to 0.24). Therefore, we considered that neither Si nor the S self-interaction have an significant effect on S partitioning for the explored compositions. With the present evidence and in the absence of a larger dataset of experiments with S at comparable P–T conditions, we infer that if the interaction between these elements has an effect on S metal–silicate partitioning it is small and within the uncertainties already produced by P–T parameterization. This conclusion can be reasonably extended to Se and Te, given the even more limited number of studies addressing these MSVE. By adding a fourth regression parameter (*d*) to the general Eq. 5, previous studies considered the effect of oxygen dissolved in liquid metal on the sulfur partitioning (*24, 65*) (Eq. 6).

(Eq. 6) $$\log_{10} \mathrm{D}_{\mathrm{i}}^{\mathrm{met-sil}} = a + \frac{b}{\mathrm{T}} + c\,\frac{\mathrm{P}}{\mathrm{T}} + \log \mathrm{X}_{\mathrm{FeO}}^{\mathrm{sil}} - \log C_s + d \log\left(1 - \mathrm{X}_{\mathrm{O}}^{\mathrm{met}}\right)$$

We found negligible effect of oxygen on the partitioning of either sulfur, selenium or tellurium (Table S4). Therefore, as in Eq. 5, we present the parameters obtained to predict behavior of S, Se, and Te as a function of P, T, $\mathrm{X}_{\mathrm{FeO}}^{\mathrm{sil}}$, and C_s,. (Table S4, Eq. 7)

(Eq. 7) $\log_{10}D_{S}^{met-sil} = (-9.00 \pm 1.36) + \frac{(14530 \pm 2784)}{T} + (220.27 \pm 46.36)\,\frac{P}{T} + \log X_{FeO}^{sil} - \log C_s$

$$\log_{10}D_{Se}^{met-sil} = (-10.31 \pm 1.20) + \frac{(18130 \pm 2601)}{T} + (233.66 \pm 39.70)\,\frac{P}{T} + \log X_{FeO}^{sil} - \log C_s$$

$$\log_{10}D_{Te}^{met-sil} = (-12.57 \pm 1.54) + \frac{(24510 \pm 3394)}{T} + (278.12 \pm 49.74)\,\frac{P}{T} + \log X_{FeO}^{sil} - log\, C_s$$

Experimental studies compilation and parametrization of $\boldsymbol{Log\ D_i^{met-sil}}$

To parameterize the partitioning of S, Se, and Te as a function of pressure and temperature (Eq. 5), we considered studies which equilibrated metal and silicate at high pressure and temperature (*9, 24, 36, 65–69, 78, 86, 10, 95–100*). Amongst them, we selected experiments based on 5 criteria: (I) capsule choice, (II) S concentration in the metal phases, (III) redox conditions, and (IV) experimental P (and T) conditions. For (I), experiments performed in containers that do not induce volatile saturation conditions were chosen – that is, performed in DAC or with MgO capsules. (II) S concentrations were considered only below 18 wt.% S, since S self-interaction can play a role at larger concentrations, while at these concentrations it does not have a significant effect (see above). Furthermore, such large S concentrations are not expected in Earth's core composition (*8, 25*). (III) We selected experiments which have a similar oxygen fugacity to Earth's magma ocean since sulfur partitioning strongly changes at oxygen fugacities lower than ΔIW-4 (ref. *9*); and (IV) we set the pressure range to be up to 65 GPa as the equilibration pressure of a deep magma ocean has been proposed to be 40–60 GPa (*10–15*). Experiments selected for S, and Se and Te compilations are presented on Table S2 and Table S3 respectively. Due to the importance of experiments such as DH15 and DH17 stated on the main discussion, and the underrepresentation of relevant experiments with S, Se, and Te at relevant P–T conditions, we weighed the model on these experiments by replicating them in the regression in order to adapt it to observables. The model shows well agreement with experimental results (Fig. 3). When regressing parameters accounting for the presence of oxygen in the metal, we only used experiments in which it was measured and reported. In this case, only few of the selected experiments can be considered for S (n=11, Table S2). A very limited number of studies were performed with Se and Te, and even fewer report $X_O^{Met.}$ (n=5, Table S3). Due to the relevance of Se and Te in this work and the limited dataset with reported oxygen content in metal, we found no statistical relevance of this fourth parameter (*d*). In Fig. S11 we report the predicted partition coefficients for different temperatures using the parameters in Table S4.

Earth's accretion and core segregation model

Based on both, cosmochemical and astrophysical constraints, Earth should have accreted from planetary embryos which may have increased in size, reaching masses comparable with those of Mars and the Moon (*30, 101*). Such impacts released enough energy to produce a global magma ocean, allowing metal–silicate segregation at depth (*13, 29*), presumably with continuous core segregation and sufficient time for complete equilibration at each step of accretion (*102–105*). This leads to an increasing core–mantle equilibration temperature and pressure, with a compositionally variable accreted material (*15, 33*) that influences oxygen fugacity changes in the mantle (*29*).

In our model, we discretized Earth's accretion in steps of differentiated bodies of 0.1% Earth's mass ($\delta m_i = 0.001$) until reaching 80% of accretion ($m_i = 0.8$) where larger bodies are incorporated ($\delta m_i = 0.02$). Earth's buildup is completed with a giant, Moon-forming impact ($\delta m_i = 0.095$) (*106–108*), as well as the late addition of 0.5% mass to evaluate the effect of a possible late veneer (*3, 16, 21*). At each step, as they are incorporated to Earth, metal–silicate equilibration occurred. This becomes less efficient for the giant impact, following magma ocean fluid dynamics simulations (*105, 109*).

We calculated at each step *i* of accretion the partition coefficient of the S, Se, Te, following our thermodynamical parametrization (Eq. 7). Pressure and temperature of equilibration ($P_{(i)}$ and $T_{(i)}$, respectively) were calculated as follows: $P_{(i)}$is associated to the final core–mantle boundary (CMB) pressure according to Eq. 8.

(Eq. 8) $$P_{(i)} = P_{CMB}\, f_i^{2/3}\, f_p$$

Where f_i is the accumulative mass fraction on Earth, f_p represents the core–mantle proportion of 0.41, and the P_{CMB} is 135 GPa. As defined in Eq. S10 of Badro et al. (*110*), the equilibration temperature ($T_{(i)}$) is an average of different calculated liquidus of peridotites (*61, 62*) (Eq. 9).

(Eq. 9) $T_{(i)} = 0.5 \left[2022 + 54.21\, P_{(i)} - 0.34\, P_{(i)}^2 + 9.0747 \cdot 10^{-4} P_{(i)}^3 + 1940 \left(\frac{P_{(i)}}{29} + 1\right)^{\frac{1}{1.9}}\right]$

$P_{(i)}$ and $T_{(i)}$ as a function of accreted mass are presented in fig. S12. FeO fraction was either considered constant (log fO_2 = -2.3 ΔIW) or varied linearly by starting from a reduced (log fO_2 = -4 ΔIW) (*29*) or from oxidized mantle (log fO_2 = -1.5 ΔIW) and reaching the current FeO estimation for the mantle (*111*) (paths A-D, Fig S2). Sulfide capacity was considered constant throughout the accretion process, according to a primitive pyrolitic composition (log C_s = -5.372, ref. *24*). Errors on the partition coefficients were calculated in a bootstrap, Monte-Carlo-like approach, where *a*, *b*, and *c* random values were picked within one σ (Table S4) to calculate $D_i^{\text{met-sil (MC}_i)}$. This process was iterated >100,000 times at each step of accretion in order to determine the uncertainty of the model (Fig. S13, shaded areas on Fig. 3).

At each step of the accretion process, we performed mass balance calculation to track S, Se, and Te abundances in Earth's core and mantle. Concentration of element *i* in the core and mantle (C_i^C or C_i^M, respectively) are calculated considering Earth's core and mantle fraction (f^C = 0.323 and f^M = (1-f^C), respectively) and mass (M) (Eq. 10).

(Eq. 10) $$C_i^{BE}\, M_i^{\text{BE}} = C_i^{BE}\, M_i^{\text{BE}}\, f^C + C_i^{BE}\, M_i^{\text{BE}} f^M$$

As impactors become larger (in our model, $\delta M_i^{IMP} > 2\%$), efficiency of equilibration is thought to decrease (*112, 113*). We therefore consider the efficiency degree of equilibration accounting for estimations of the silicate and metal density, and the partition coefficient of S, Se, and Te to determine the likelihood of these elements diffusing out of the accreting core during its fragmentation and assimilation in the magma ocean (Eq. 11) (*109*).

(Eq. 11) $$\epsilon_i = \frac{k}{1+(\text{D}_i^{\text{met-sil}}/\Delta)}$$

Where ϵ_i is the equilibration efficiency of an element *i*, k is the fraction of the accreted core equilibrating with Earth's mantle, where 1-k fraction merges with Earth's core. This is k=1

for smaller impactors and k=0.5 for the giant impact. Δ is the metal dilution, which is defined as in Eq. 12.

(Eq. 12)
$$\Delta = \frac{\rho_{\text{sil}}}{\rho_{\text{met}}}\left[\left(1+\alpha\frac{z}{r_0}\right)^3 - 1\right]$$

With ρ referring to silicate and metal densities, α to the entrainment coefficient, z being related to the depth of equilibration, and r_0 to the radius of the blobs (*109*). Therefore, the mass conservation of the accreted material throughout the stepwise accretion model would be calculated for the mantle (Eq. 13) and the core (Eq. 14) (*31, 105*).

(Eq. 13)
$$\frac{d}{dt}\left[(1-f^C)M^{BE}C_i^M\right] = \left[(1-f^C)C_i^{M\text{-}IMP} + \epsilon_i f^C\left(C_i^{C-IMP} - D_i C_i^M\right)\right]\frac{dM}{dt}$$

(Eq. 14)
$$\frac{d}{dt}\left[f^C M^{BE} C_i^C\right] = \left[(1-\epsilon_i)f^C C_i^{C-IMP} + \epsilon_i f^C D_i C_i^M\right]\frac{dM}{dt}$$

Consequently, Eqs. 13 and 14 are calculated at each step of accretion, and adds up to the accumulated concentration at step *i*, considering therefore the inefficiently equilibrated mass of the accreting core.

In terms of absolute abundances, each differentiated impactor was made out of a bulk Earth MSVE-like concentration: 6350 ppm S, 2.7 ppm Se, 0.30 ppm Te (*4–7*). To account for different proposed scenarios, this type of material was either added throughout the accretion process (homogeneous model, e.g., *32*) or in the last stages of Earth's accretion and core formation, after 80% Earth's mass accreted (heterogeneous model, e.g., *33–37*). The final mantle contains 146^{+93}_{-57} ppm S, 69^{+38}_{-25} ppb Se, and 5^{+4}_{-2} ppb Te when considering a homogeneous volatile accretion model. For a heterogeneous volatile accretion model, they are similar within uncertainties, with 197^{+125}_{-77} ppm S, 72^{+40}_{-26} ppb Se, and 5^{+4}_{-2} ppb Te. The sensitivity of the model was tested by varying k values. Between 80 to 90% of accreted Earth's mass, k was decreased to 0.5, and during the giant impact, it was set to k=0.1. This produced a mantle containing 147^{+93}_{-57} ppm S, 69^{+38}_{-25} ppb Se, and 5^{+4}_{-2} ppb Te for a homogenous delivery, and 162^{+125}_{-77} ppm S, 50^{+40}_{-26} ppb Se, and 3^{+4}_{-2} for a heterogeneous model of volatile delivery. Therefore, the final mantle abundances remain the same within uncertainties independently of k values.

The composition of the Earth's mantle (Fig. 4) and the core (fig. S4) show consistent results, matching both peridotite- or MORB-derived compositional models. As a reminder, peridotite models suggest the Earth's mantle contains 217±38 ppm S, 77±38 ppb Se, and 10±5 ppb Te (*4–7*). In contrast, MORB-derived models, the BSE is composed of 185±21 ppm S, 49±11 to 80 ±17 ppb Se, and 3±1 to 11±2 ppb Te (*42*). Note that, by using bulk Earth abundances allow to avoid unconstrained effects such as evaporation processes (*38, 39, 114–116*). However, we also examined a simple approximation to this effect performed on ab initio calculation studies (*39*). There, metal–silicate distribution of S, Se, and Te were assumed to be 70, by necessity similar to one another, $\log D_{S}^{met-sil} \approx \log D_{Se}^{met-sil} \approx \log D_{Te}^{met-sil}$, which is confirmed here (see main text). Additionally, they considered similar condensation behavior and vapor–silicate partitioning, $\log D_{S}^{vap-sil} \approx \log D_{Se}^{vap-sil} \approx \log D_{Te}^{vap-sil}$, of all three elements (*39*). By taking a mixture of volatile-bearing materials: enstatite chondrites, OC, and CI (*28*), we observe that BSE abundances can be matched (Fig. S6). Therefore, current abundances can be explained by metal–silicate segregation in first order without strictly requiring secondary processes (see below) (*40, 41*) or late addition after core segregation (see main text, *2, 16*).

Negligible effect of sulfide segregation and iron disproportionation on S, Se, and Te abundance

S, Se, and Te budget in BSE can be additionally affected by secondary processes. Sulfide segregation (*aka* Hadean matte) may favor chalcophile elements extraction (including HSE) and segregation to the core (*40, 80, 117, 118*). Experimental studies suggest that no scenario would produce the saturated mantle needed from sulfur concentration at sulfide saturation (SCSS) (*80, 117, 119, 120*) (Fig. S14) and selenide content at selenide saturation (*121*) calculations. We can assume that Te behaves similarly. Very specific conditions could account for sulfide segregation. For example, if solidification of the magma ocean produces a basal magma ocean enriched in incompatible elements (*122*), this could increase S concentration towards SCSS. Given that mantle and core abundances can be matched without calling for sulfide saturation (Fig. 4, S4, S14), that SCSS is very high at the end of Earth's accretion compared to its S concentration (*119, 120*) (Fig. S14), and the constrained scenarios that could produce sulfide segregation. We argue that the Hadean Matte may have not occurred or, at least, it is not requested to explain the composition of Earth's mantle.

S, Se, and Te may also be affected by FeO disproportionation into Fe^0 and Fe_2O_3 at lower mantle depths (*41*). The formation of metallic Fe that equilibrates with the surrounding silicate melt would lead to the partitioning of S, Se, and Te into the metal phase, making it a carrier of these elements into Earth's core. This "self-oxidation" event of Earth's mantle potentially leads to the depletion of S, Se, and Te from the BSE. This has been recently proposed as a factor for Pt depletion in BSE (*31*). From low pressure and temperature experiments, iron disproportionation would be particularly effective at ~ 25 GPa (*123*), which is after 20% of Earth's mass accretion (fig. S12), where log $\mathrm{D}_{\mathrm{S,Se,Te}}^{\mathrm{met-sil}}$ are already very close to the core–mantle distribution (Fig. 3), with Te at slightly higher values yet overlapping within errors with S and Se. Hence chondritic-like proportions would still be preserved. Mass balance calculations indicate that only an extremely efficient case, where 3 wt.% Fe subtraction from the mantle occurs (*41, 124*), would subtract 107 ppm, 51 ppb, and 4 ppb of S, Se, and Te, respectively (accounting for $\log D_{S,Se,Te}^{\mathrm{met-sil}} \approx 1.9$ from DH15 and DH17) However, recent in situ experiments show that disproportionation at direct conditions of a deep magma ocean is substantially less effective (*124*), suggesting that, for the same pressure, less Fe^{3+}/Fe total is produced by this effect than predicted from low pressure data (*123*). This means that, if these processes were to occur, S, Se, and Te budget in the BSE would remain mostly unaffected.

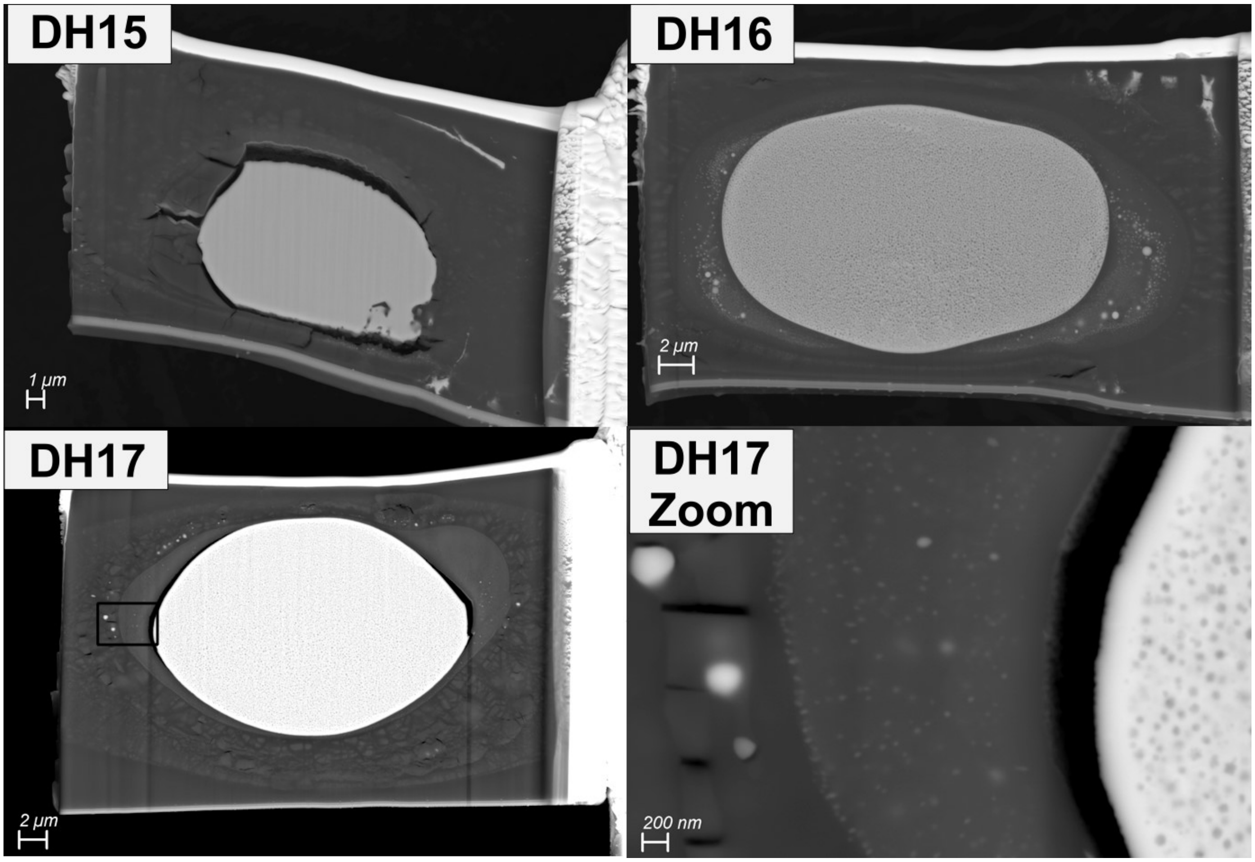

Fig. S1.

Backscattering electron image on run products, presenting metal and silicate melts, as well as unreactive silicate glasses. On DH17, the black rectangle indicates the expanded region, where metal inclusions are observed. Their homogeneous distribution may indicate they are formed upon quenching (*14, 24, 65, 77*).

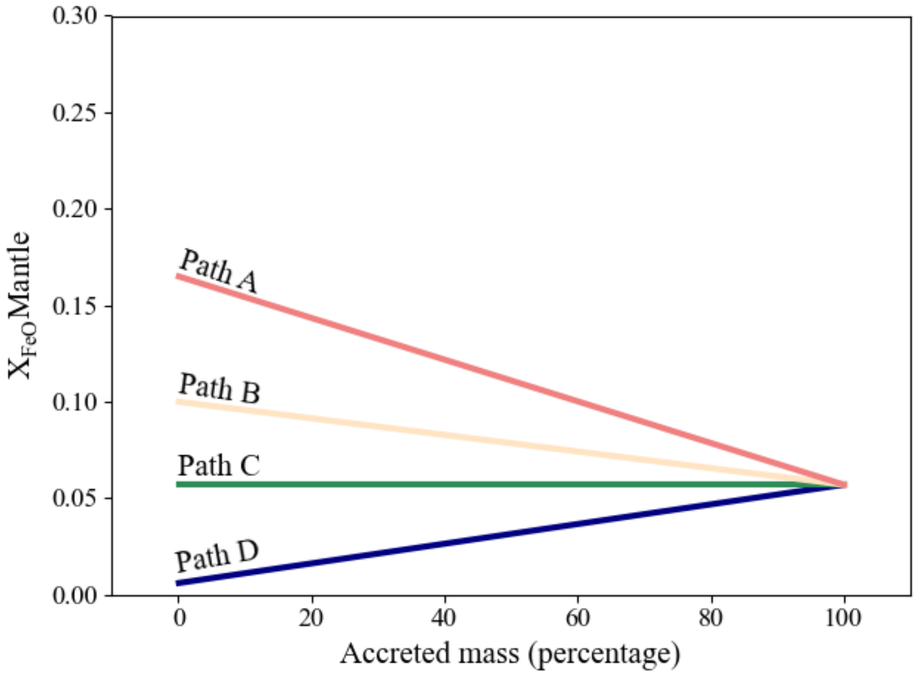

Fig. S2.

Oxygen fugacity paths tested on the accretion models. We tested two oxidized initial magma oceans based on seismological and geochemical constraints (*13, 110*) (initial log fO_2 ΔIW-1.5 and -1.9, paths A and B, respectively), a magma ocean of very reduced initial oxygen fugacity conditions (*29*) (log fO_2 ΔIW-4.5, path D), as well as a constant oxygen fugacity path at log fO_2 ΔIW-2.3 (path C).

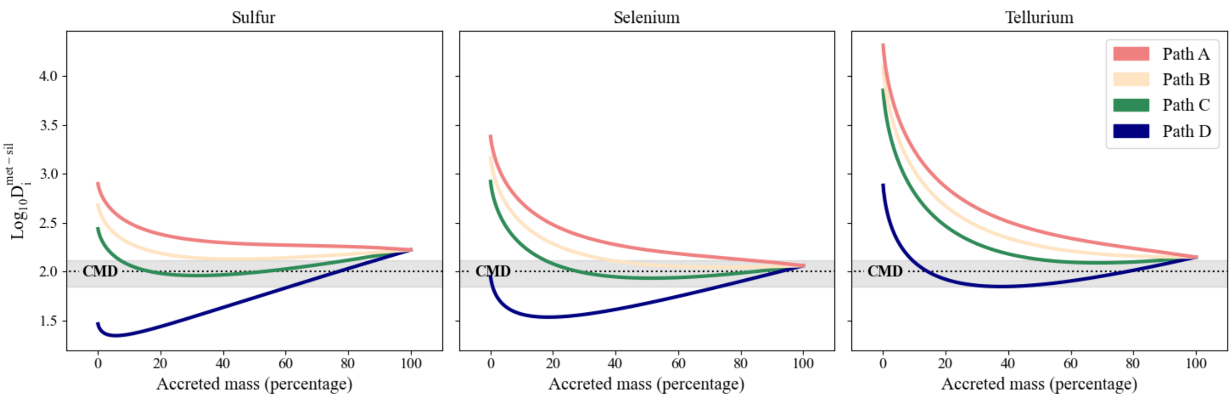

Fig. S3.

Calculated partition coefficient variation (Eq. 7) for all initial oxygen fugacity tested in this study. Color coded as in fig. S2, where magma oceans with initial log fO_2 of ΔIW-1.5, ΔIW-1.9, ΔIW-2.3, and ΔIW-4.5 are paths A to D, respectively. For simplicity, errors are not reported but are in the same orders of magnitude as in Fig. 3. CMD: Core–mantle distribution.

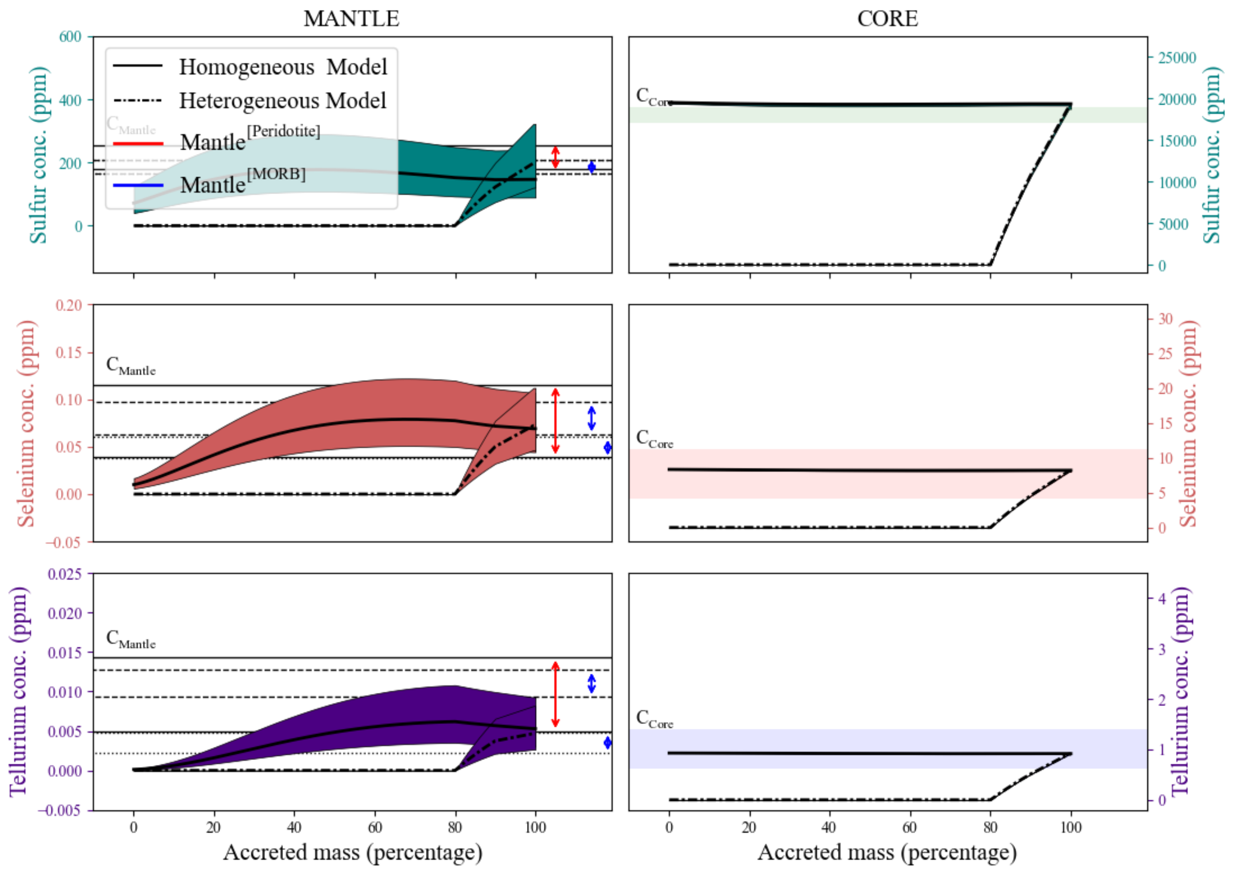

Fig. S4.

Elemental abundance of S, Se, and Te in Earth's mantle and core as a function of accreted mass to Earth at constant oxygen fugacity conditions (path C). Mantle's abundances are highlighted as in Fig. 4 (*4–7, 42*), while core composition is highlighted as shaded areas (*4– 6, 8*).

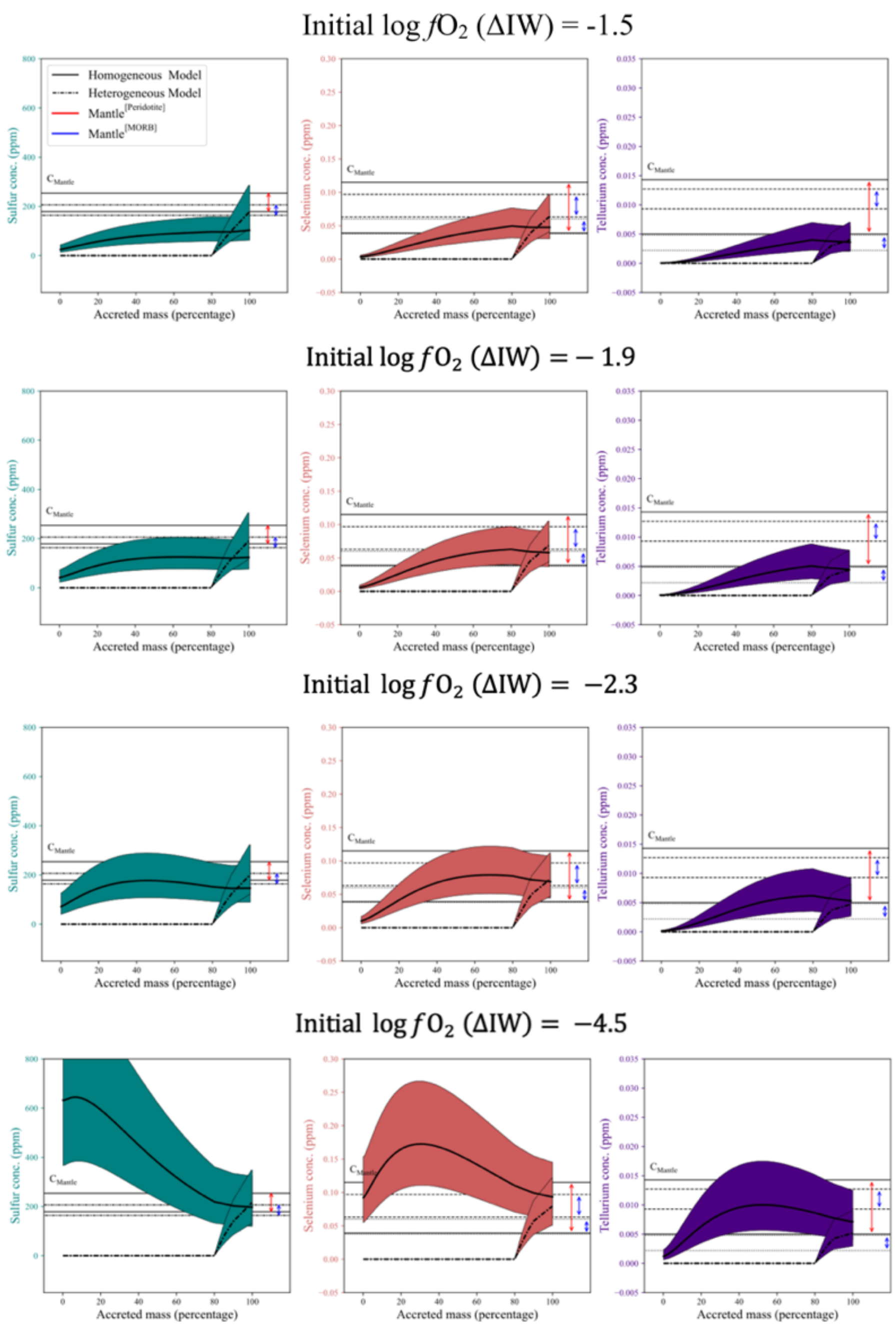

Fig. S5.

Elemental abundances of S, Se, and Te in the mantle (as in Fig. 4) at all initial $\log fO_2$ conditions explored on this study (paths A to D) (Fig. S2, S3). Red and blue arrows indicate upper and lower bounds for Earth's mantle composition from peridotite (*4*–*7*) and MORB (*42*) estimates, respectively. All models match Earth's mantle composition.

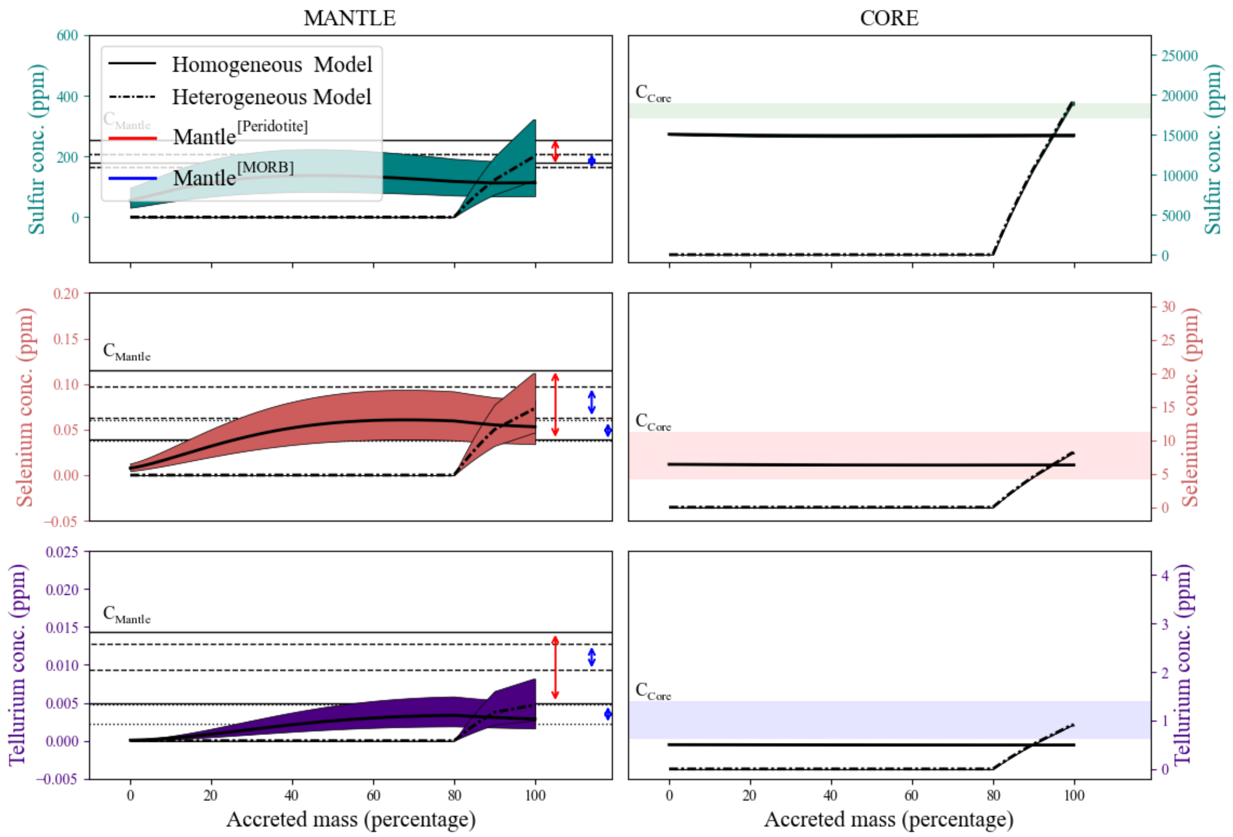

Fig. S6.

As in Figure S4, Elemental abundances of S, Se, and Te as a function of Earth's accreted mass at constant log fO_2 ΔIW-2.3 (path C). Here, considering building blocks of a EC-OC-CC mixture (*28*) and the delivery of volatiles after 90% of evaporation and loss to space (*38, 39*). Earth's mantle composition from peridotite (*4–7*) and MORB (*42*) estimates can be matched.

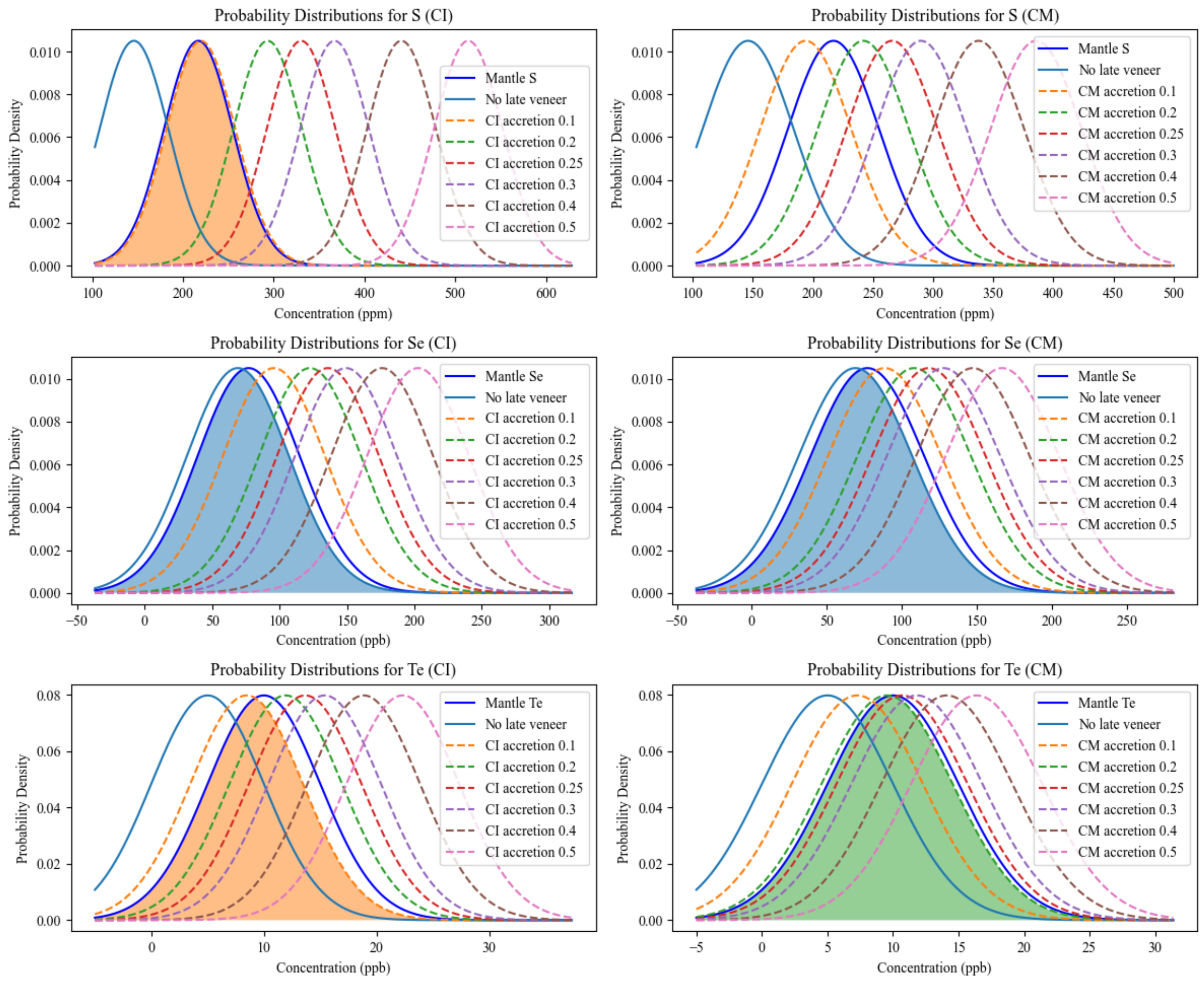

Fig. S7.

Probability density functions for CM- and CI-like late veneer increments after volatile accretion on the main stage of Earth's formation (Table S6) (dashed lines), Earth's mantle compositions from peridotite models (continuous blue line, *4–7*), as well as model results without late veneer contribution (continuous grey line). Shaded areas indicate cases where there is an overlap higher than 1σ and statistically similar mean values with p value ≥ 0.05.

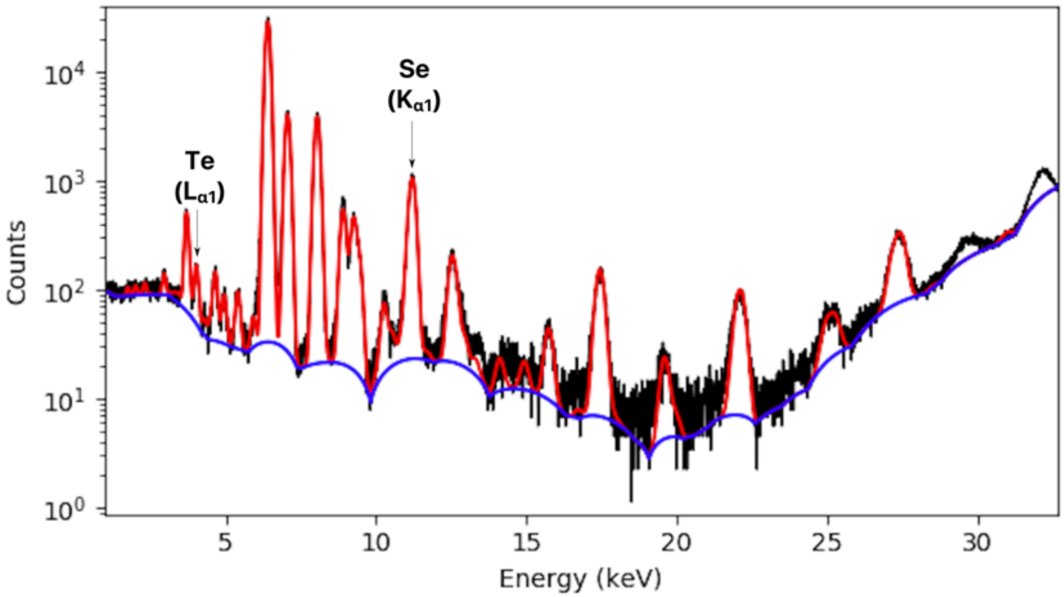

Fig. S8.
X-ray fluorescence spectrum fitting using PyMCA (*72*) on DH14. Black curves are collected spectra, red and blue curves are the fitting and the background, respectively. We labelled peaks from where Se and Te were quantified. EPMA analyses were used to normalize for matrix composition.

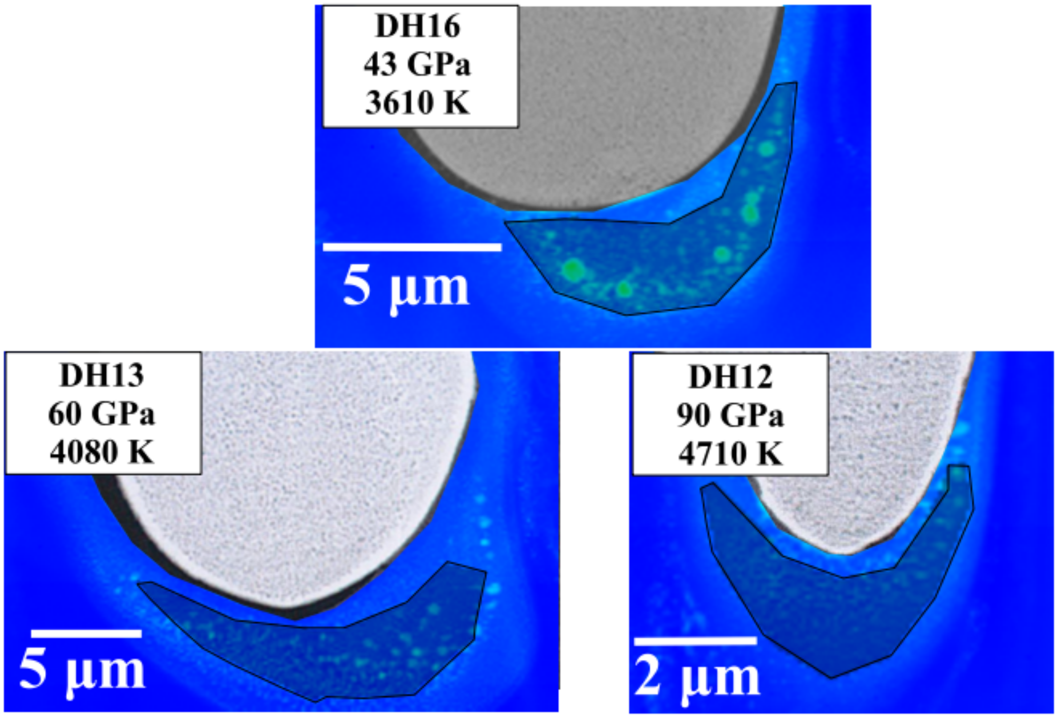

Fig. S9.

Stacked nano-resolved X-ray fluorescence maps collected on DH12, DH13, and DH16. The ROIs (shaded areas) were selected integrating metal inclusions.

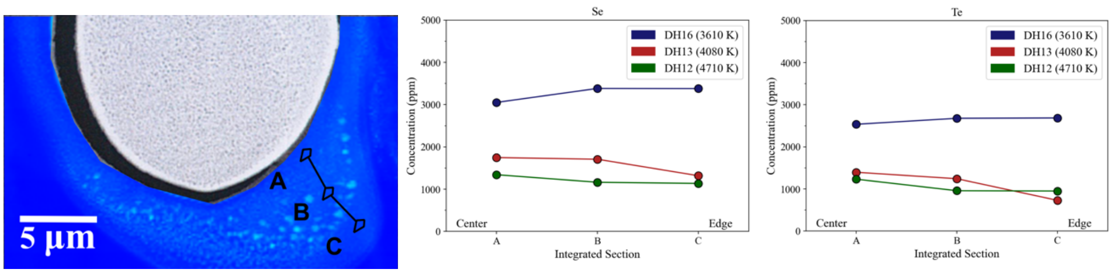

Fig. S10.

On the left, profile collection on stacked nano-resolved X-ray fluorescence map of DH13 to show equilibrium in the silicate composition. On the center and right, Se and Te profile composition for DH16, DH13, and DH12, respectively. The experiments with lowest and highest temperature and pressure conditions are presented, as well as one intermediate. No significant difference is observed between the collections. Smaller compositional differences towards the edge could be associated to ROI selection partially overlapping unreacted (Se, Te)-free peridotitic glass. Errors are smaller than symbols.

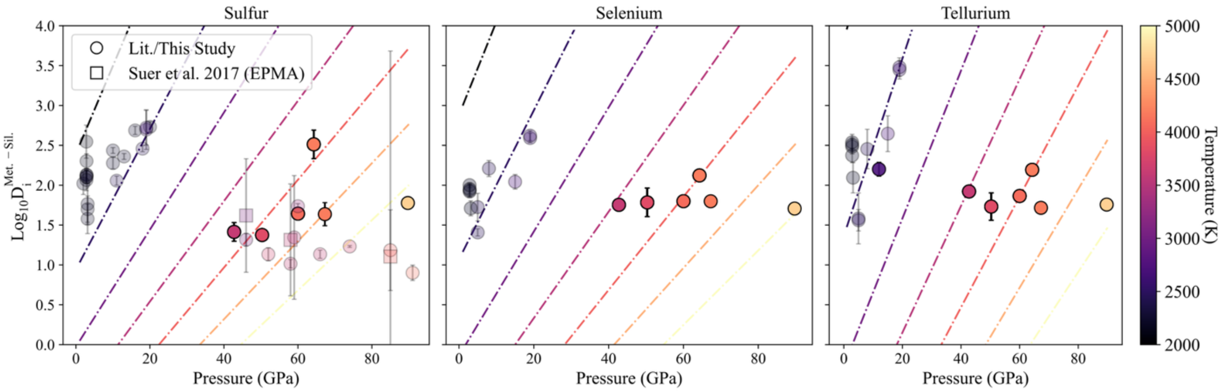

Fig. S11.

Partition coefficient presented in logarithmic units as a function of pressure for S, Se, and Te. We present our results, together with relevant experiments of previous studies (Lit., semitransparent symbols, see supplementary text) (Table S2, S3), where in squares are indicated similar type of experiments measured with the same analytical technique employed here (*24*). The dashed lines are modelled partitioning curves at constant temperatures. Experiments and models are color-coded according to their respective equilibration temperatures. The modelled partition coefficients agree well with the measured results.

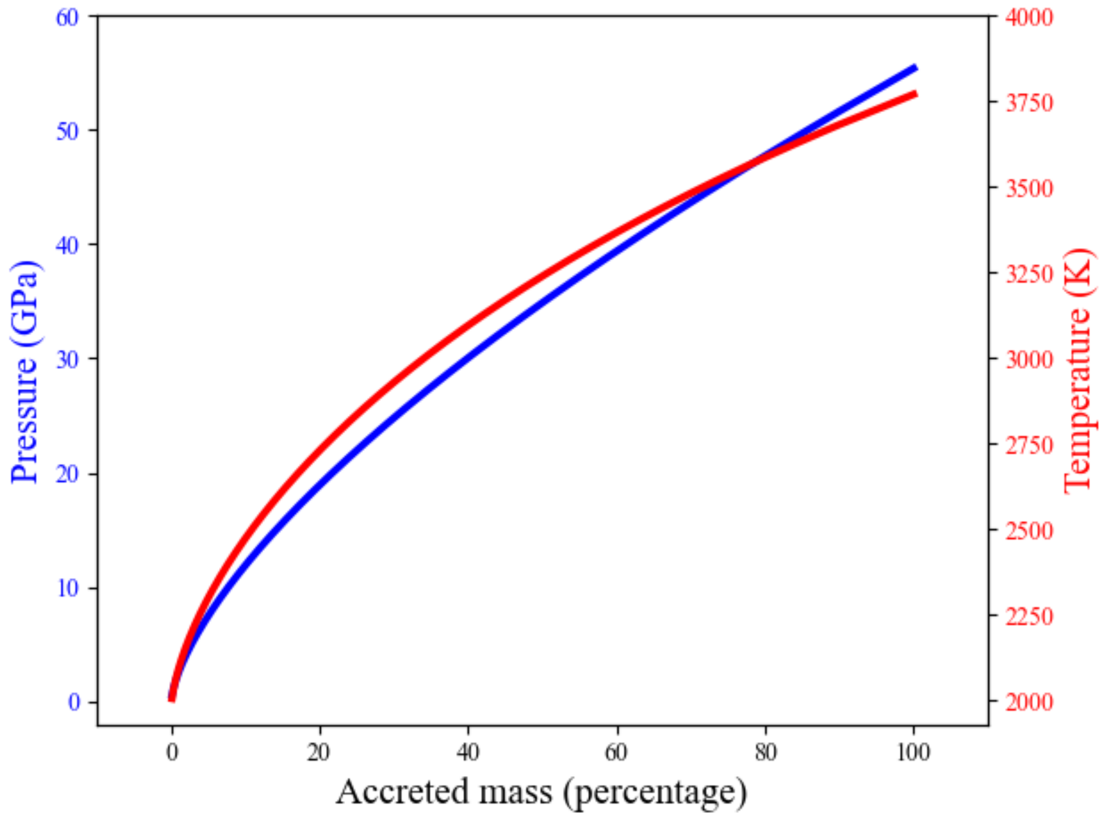

Fig. S12.
Pressure and temperature evolution curves of metal–silicate equilibration conditions in blue and red, respectively. Final conditions at full accreted Earth equilibration indicate a deep magma ocean of ~ 55 GPa and 3750 K, similar to previous studies (e.g., *13, 67*).

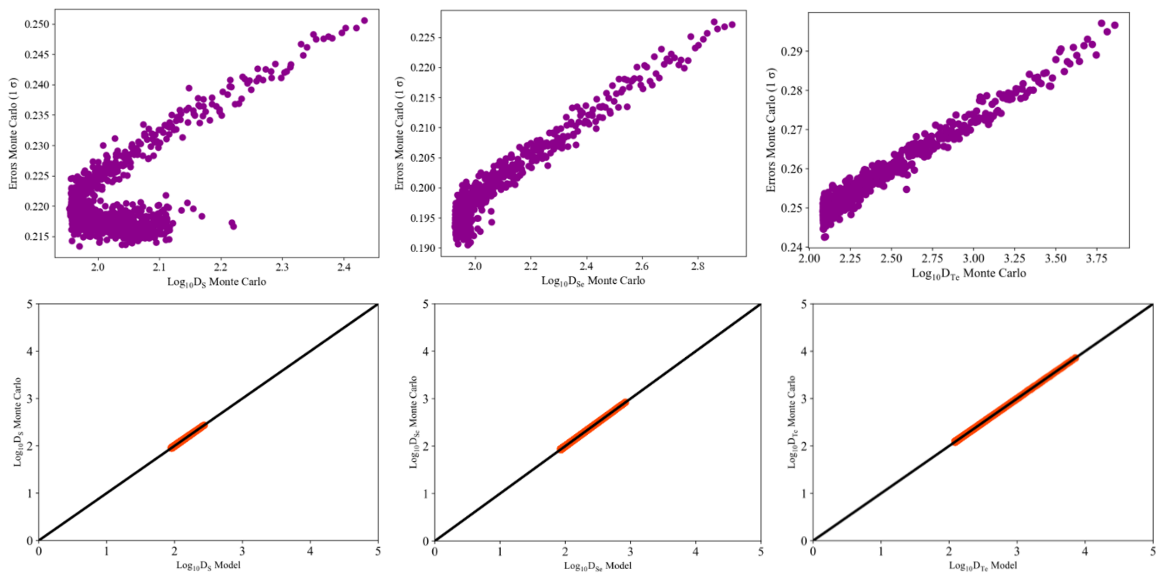

Fig. S13.

Comparison between predicted log D_i values on the accretion model and the errors predicted by a Monte Carlo-like approach with >100,000 iterations (S, Se, and Te, from left to right). On the upper row, we present the predicted vs modelled values. On the lower row, the errors vs Monte Carlo-estimated log D.

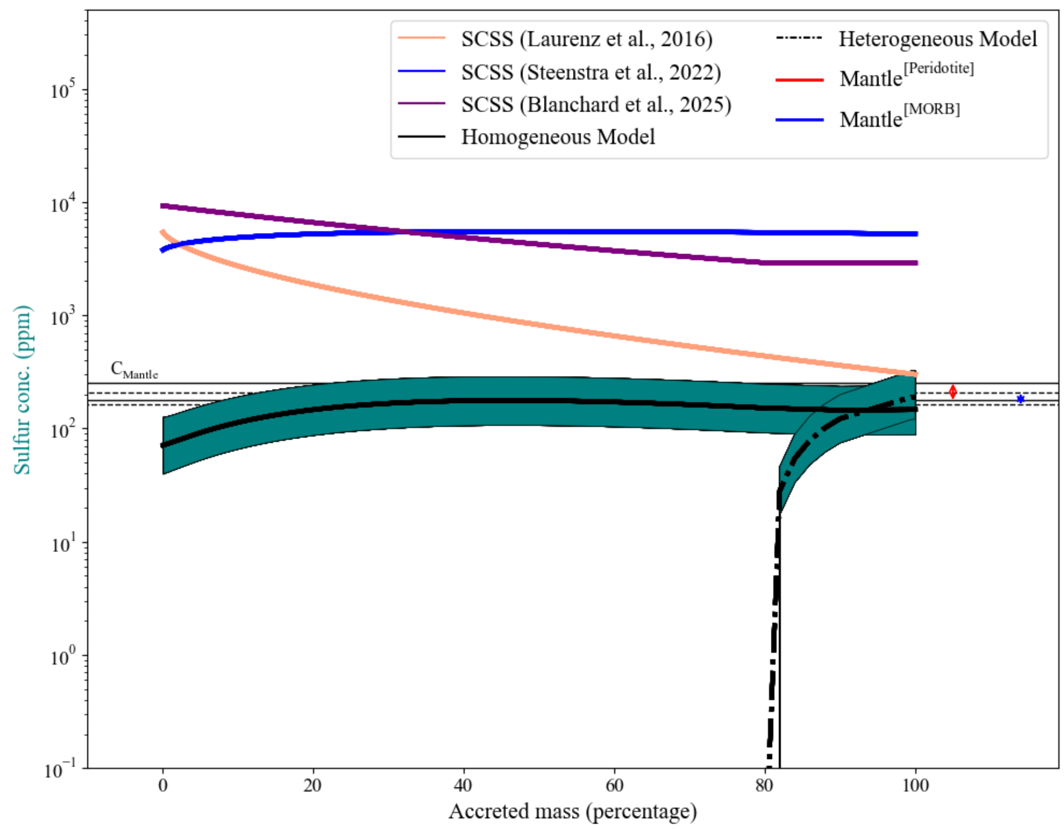

Fig. S14.

Sulfur concentration in ppm (presented in logarithmic units) as a function of accreted mass to Earth. Sulfur concentration at sulfide saturation (SCSS) calculated after Laurenz et al. (*117*), Steenstra et al., (*119*), and Blanchard et al. (*120*): Laurenz et al's model is an extrapolation from low pressure experimental data, whereas Steenstra et al's and Blanchard et al.'s models are calibrated from experiments at similar P–T conditions with basaltic and pyrolitic compositions, respectively. No saturation conditions should be observed in the presence of S, Se, and Te. Therefore, the 'Hadean matte' (*40, 118*) is not requested to explain these MSVE concentrations

List of Supplementary Table captions

Table S1. Experimental conditions and EPMA and Nano-XRF compositional results on the run products.

Table S2. Experiments compiled for Sulfur multilinear regression.

Table S3. Experiments compiled for Selenium and Tellurium multilinear regression.

Table S4. Parameters and coefficients obtained by multilinear regression on compiled data.

Table S5. Meteorite composition (in ppm) for Late Veneer input in accretion model.

Table S6. t-test and Bayesian inference analyses results for a late veneer increment.

Table S7. Starting Material (Standard) composition. Modified from LRMS34 (ref. 9).

Table S8. EPMA and Nano-XRF composition obtained for E595 internal standard.

REFERENCES

1. K. Lodders, Solar System abundances and condensation temperatures of the elements. *Astrophys. J.* **591**, 1220 (2003).

2. C. L. Chou, Fractionation of siderophile elements in the Earth's upper mantle. *Proc. Lunar Planet. Sci. Conf.* **1**, 219–230 (1978).

3. R. J. Walker, Highly siderophile elements in the Earth, Moon and Mars: Update and implications for planetary accretion and differentiation. *Geochemistry* **69**, 101–125 (2009).

4. W. F. McDonough, S. S. Sun, The composition of the Earth. *Chem. Geol.* **120**, 223–253 (1995).

5. W. F. McDonough, in *Treatise on Geochemistry*, H.D. Holland, K. K. Turekian, Eds. (Elsevier, ed. 2, 2003), pp. 547–568.

6. H. Palme, H. O'Neill, in vol. 3 of *Treatise on Geochemistry*, H.D. Holland, K. K. Turekian, Eds. (Elsevier, ed. 2, 2014), pp. 1–39.

7. J. P. Lorand, A. Luguet, Chalcophile and siderophile elements in mantle rocks: Trace elements controlled by trace minerals. *Rev. Mineral. Geochemistry* **81**, 441–488 (2016).

8. G. Dreibus, H. Palme, Cosmochemical constraints on the sulfur content in the Earth's core. *Geochim. Cosmochim. Acta* **60**, 1125–1130 (1996).

9. L. Rose-Weston, J. M. Brenan, Y. Fei, R. A. Secco, D. J. Frost, Effect of pressure, temperature, and oxygen fugacity on the metal-silicate partitioning of Te, Se, and S: Implications for Earth differentiation. *Geochim. Cosmochim. Acta* **73**, 4598–4615 (2009).

10. B. J. Wood, J. Wade, M. R. Kilburn, Core formation and the oxidation state of the Earth: Additional constraints from Nb, V and Cr partitioning. *Geochim. Cosmochim. Acta* **72**, 1415–1426 (2008).

11. E. Cottrell, M. J. Walter, D. Walker, Metal-silicate partitioning of tungsten at high pressure and temperature: Implications for equilibrium core formation in Earth. *Earth Planet. Sci. Lett.* **281**, 275–287 (2009).

12. M. A. Bouhifd, A. P. Jephcoat, Convergence of Ni and Co metal-silicate partition coefficients in the deep magma-ocean and coupled silicon-oxygen solubility in iron melts at high pressures. *Earth Planet. Sci. Lett.* **307**, 341–348 (2011).

13. J. Siebert, J. Badro, D. Antonangeli, F. J. Ryerson, Metal-silicate partitioning of Ni and Co in a deep magma ocean. *Earth Planet. Sci. Lett.* **321**, 189–197 (2012).

14. R. A. Fischer, Y. Nakajima, A. J. Campbell, D. J. Frost, D. Harries, F. Langenhorst, N. Miyajima, K. Pollok, D. C. Rubie, High pressure metal-silicate partitioning of Ni, Co, V, Cr, Si, and O. *Geochim. Cosmochim. Acta* **167**, 177–194 (2015).

15. D. C. Rubie, D. J. Frost, U. Mann, Y. Asahara, F. Nimmo, K. Tsuno, P. Kegler, A. Holzheid, H. Palme, Heterogeneous accretion, composition and core-mantle differentiation of the Earth. *Earth Planet. Sci. Lett.* **301**, 31–42 (2011).

16. Z. Wang, H. Becker, Ratios of S, Se and Te in the silicate Earth require a volatile-rich late veneer. *Nature* **499**, 328–331 (2013).

17. K. K. Turekian, S. P. Clark Jr., Inhomogeneous accumulation of the earth from the primitive solar nebula. *Earth Planet. Sci. Lett.* **6**, 346–348 (1969).

18. K. Kimura, R. S. Lewis, E. Anders, Distribution of gold and rhenium between nickel-iron and silicate melts: Implications for the abundance of siderophile elements on the Earth and Moon. *Geochim. Cosmochim. Acta* **38**, 683–701 (1974).

19. H. Wänke, Constitution of terrestrial planets. *Philos Trans A Math Phys Eng Sci* **303**, 287–302 (1981).

20. M. I. Varas-Reus, S. König, A. Yierpan, J. P. Lorand, R. Schoenberg, Selenium isotopes as tracers of a late volatile contribution to Earth from the outer Solar System. *Nat. Geosci.* **12**, 779–782 (2019).

21. F. Albarede, Volatile accretion history of the terrestrial planets and dynamic implications. *Nature* **461**, 1227–1233 (2009).

22. D. Andrault, S. Petitgirard, G. Lo Nigro, J. L. Devidal, G. Veronesi, G. Garbarino, M. Mezouar, Solid-liquid iron partitioning in Earth's deep mantle. *Nature* **487**, 354–357 (2012).

23. G. K. Pradhan, G. Fiquet, J. Siebert, A. L. Auzende, G. Morard, D. Antonangeli, G. Garbarino, Melting of MORB at core-mantle boundary. *Earth Planet. Sci. Lett.* **431**, 247–255 (2015).

24. T.-A. Suer, J. Siebert, L. Remusat, N. Menguy, G. Fiquet, A sulfur-poor terrestrial core inferred from metal-silicate partitioning experiments. *Earth Planet. Sci. Lett.* **469**, 84–97 (2017).

25. K. Hirose, B. J. Wood, L. Vočadlo, Light elements in the Earth's core. *Nat. Rev. Earth Environ.* **2**, 645–658 (2021).

26. C. J. Allegre, J. P. Poirier, E. Humler, A. W. Hofmann, The chemical composition of the Earth. *Earth Planet. Sci. Lett.* **134**, 515–526 (1995).

27. M. Javoy, E. Kaminski, F. Guyot, D. Andrault, C. Sanloup, M. Moreira, S. Labrosse, A. Jambon, P. Agrinier, A. Davaille, C. Jaupart, The chemical composition of the Earth: Enstatite chondrite models. *Earth Planet. Sci. Lett.* **293**, 259–268 (2010).

28. N. Dauphas, The isotopic nature of the Earth's accreting material through time. *Nature* **541**, 521–524 (2017).

29. J. Wade, B. J. Wood, Core formation and the oxidation state of the Earth. *Earth Planet. Sci. Lett.* **236**, 78–95 (2005).

30. D. C. Rubie, S. A. Jacobson, A. Morbidelli, D. P. O'Brien, E. D. Young, J. de Vries, F. Nimmo, H. Palme, D. J. Frost, Accretion and differentiation of the terrestrial planets with implications for the compositions of early-formed Solar System bodies and accretion of water. *Icarus* **248**, 89–108 (2015).

31. T. A. Suer, J. Siebert, L. Remusat, J. M. Day, S. Borensztajn, B. Doisneau, G. Fiquet, Reconciling metal-silicate partitioning and late accretion in the Earth. *Nat. Commun.* **12**, 2913 (2021).

32. K. Righter, M. J. Drake, Metal-silicate equilibrium in a homogeneously accreting Earth: New results for Re. *Earth Planet. Sci. Lett.* **146**, 541–553 (1997).

33. M. Schönbächler, R. Carlson, M. Horan, T. Mock, E. Hauri, Heterogeneous accretion and the moderately volatile element budget of Earth. *Science* **328**, 884–887 (2010).

34. B. Mahan, J. Siebert, I. Blanchard, S. Borensztajn, J. Badro, F. Moynier, Constraining compositional proxies for Earth's accretion and core formation through high pressure and high temperature Zn and S metal-silicate partitioning. *Geochim. Cosmochim. Acta* **235**, 21–40 (2018).

35. N. Braukmüller, F. Wombacher, C. Funk, C. Münker, Earth's volatile element depletion pattern inherited from a carbonaceous chondrite-like source. *Nat. Geosci.* **12**, 564–568 (2019).

36. E. Kubik, J. Siebert, B. Mahan, J. Creech, I. Blanchard, A. Agranier, S. Shcheka, F. Moynier, Tracing Earth's volatile delivery with tin. *J. Geophys. Res. Solid Earth* **126**, e2021JB022026 (2021).

37. E. Kubik, J. Siebert, I. Blanchard, A. Agranier, B. Mahan, F. Moynier, Earth's volatile accretion as told by Cd, Bi, Sb and Tl core-mantle distribution. *Geochim. Cosmochim. Acta* **306**, 263–280 (2021).

38. W. Wang, C. H. Li, J. P. Brodholt, S. Huang, M. J. Walter, M. Li, Z. Wu, F. Huang, S. J. Wang, Sulfur isotopic signature of Earth established by planetesimal volatile evaporation. *Nat. Geosci.* **14**, 806–811 (2021).

39. W. Wang, M. J. Walter, J. P. Brodholt, S. Huang, M. I. Petaev, Chalcogen isotopes reveal limited volatile contribution from late veneer to Earth. *Sci. Adv.* **9**, 1–10 (2023).

40. H. S. C. O’Neill, The origin of the Moon and the early history of the Earth—A chemical model. Part 2: The Earth. *Geochim. Cosmochim. Acta* **55**, 1159–1172 (1991).

41. D. J. Frost, C. Liebske, F. Langenhorst, C. A. McCammon, R. G. Trønnes, D. C. Rubie, Experimental evidence for the existence of iron-rich metal in the Earth’s lower mantle. *Nature* **428**, 409–412 (2004).

42. A. Yierpan, S. König, J. Labidi, R. Schoenberg, Selenium isotope and S-Se-Te elemental systematics along the Pacific-Antarctic ridge: Role of mantle processes. *Geochim. Cosmochim. Acta* **249**, 199–224 (2019).

43. M. F. Horan, R. J. Walker, J. W. Morgan, J. N. Grossman, A. E. Rubin, Highly siderophile elements in chondrites. *Chem. Geol.* **196**, 27–42 (2003).

44. T. Iizuka, T. Shibuya, T. Hayakawa, T. Yokoyama, I. Gautam, M. K. Haba, K. T. M. Ito, Y. Hibiya, A. Yamaguichi, Y. Abe, J. Aléon, C. M. O. D. Alexander, S. Amari, Y. Amelin, K. Bajo, M. Bizzarro, A. Bouvier, R. W. Carlson, M. Chaussidon, B.-G. Choi, N. Dauphas, A. M. Davis, T. Di Rocco, W. Fujiya, R. Fukai, H. Hidaka, H. Homma, G. R. Huss, T. R. Ireland, A. Ishikawa, S. Itoh, N. Kawasaki, N. T. Kita, K. Kitajima, T. Kleine, S. Komatani, A. N. Krot, M.-C. Liu, Y. Masuda, K. Motomura, F. Moynier, K. Nagashima, I. Nakai, A. Nguyen, L. Nittler, A. Pack, C. Park, L. Piani, L. Qin, S. Russell, N. Sakamoto, M. Schönbächler, L. Tafla, H. Tang, K. Terada, Y. Terada, T. Usui, S. Wada, M. Wadhwa, R. J. Walker, K. Yamashita, Q.-Z. Yin, S. Yoneda, H. Yui, A.-C. Zhang, T. Nakamura, H. Naraoka, T. Noguchi, R. Okazaki, K. Sakamoto, H. Yabuta, M. Abe, A. Miyazaki, A. Nakato, M. Nishimura, T. Okada, T. Yada, K. Yogata, S. Nakazawa, T. Saiki, S. Tanaka, F. Terui, Y. Tsuda, S. Watanabe, M. Yoshikawa, S. Tachibana, H. Yurimoto, Late fluid flow in a primitive asteroid revealed by Lu-Hf isotopes in Ryugu. *Nature* **646**, 62–67 (2025).

45. T. Yokoyama, N. Dauphas, R. Fukai, T. Usui, S. Tachibana, M. Schönbächler, H. Busemann, M. Abe, T. Yada, The elemental abundances of Ryugu: Assessment of chemical heterogeneities and the nugget effect. *Geochem. J.* **59**, 45–63 (2025).

46. M. Fischer-Gödde, B. M. Elfers, C. Münker, K. Szilas, W. D. Maier, N. Messling, T. Morishita, M. Van Kranendonk, H. Smithies, Ruthenium isotope vestige of Earth's pre-late-veneer mantle preserved in Archaean rocks. *Nature* **579**, 240–244 (2020).

47. J. Labidi, J. Farquhar, C. O. D. Alexander, D. L. Eldridge, H. Oduro, Mass independent sulfur isotope signatures in CMs: Implications for sulfur chemistry in the early Solar System. *Geochim. Cosmochim. Acta* **196**, 326–350 (2017).

48. J. Labidi, S. König, T. Kurzawa, A. Yierpan, R. Schoenberg, The selenium isotopic variations in chondrites are mass-dependent; Implications for sulfide formation in the early Solar System. *Earth Planet. Sci. Lett.* **481**, 212–222 (2018).

49. U. Mann, D. J. Frost, D. C. Rubie, H. Becker, A. Audétat, Partitioning of Ru, Rh, Pd, Re, Ir and Pt between liquid metal and silicate at high pressures and high temperatures—Implications for the origin of highly siderophile element concentrations in the Earth's mantle. *Geochim. Cosmochim. Acta* **84**, 593–613 (2012).

50. L. Piani, Y. Marrocchi, T. Rigaudier, L. G. Vacher, D. Thomassin, B. Marty, Earth's water may have been inherited from material similar to enstatite chondrite meteorites. *Science* **369**, 1110–1113 (2020).

51. I. Blanchard, D. C. Rubie, E. S. Jennings, I. A. Franchi, X. Zhao, S. Petitgirard, N. Miyajima, S. A. Jacobson, A. Morbidelli, The metal-silicate partitioning of carbon during Earth's accretion and its distribution in the early Solar System. *Earth Planet. Sci. Lett.* **580**, 117374 (2022).

52. D. S. Grewal, R. Dasgupta, C. Sun, K. Tsuno, G. Costin, Delivery of carbon, nitrogen, and sulfur to the silicate Earth by a giant impact. *Sci. Adv.* **5**, eaau3669 (2019).

53. D. S. Grewal, R. Dasgupta, A. K. Holmes, G. Costin, Y. Li, K. Tsuno, The fate of nitrogen during core-mantle separation on Earth. *Geochim. Cosmochim. Acta* **251**, 87–115 (2019).

54. C. R. M. Jackson, E. Cottrell, Z. Du, N. R. Bennett, Y. Fei, High pressure redistribution of nitrogen and sulfur during planetary stratification. *Geochem. Perspect. Lett* **18**, 37–42 (2021).

55. Y. Li, L. Vočadlo, T. Sun, J. P. Brodholt, The Earth's core as a reservoir of water. *Nat. Geosci.* **13**, 453–458 (2020).

56. S. Tagawa, N. Sakamoto, K. Hirose, S. Yokoo, J. Hernlund, Y. Ohishi, H. Yurimoto, Experimental evidence for hydrogen incorporation into Earth's core. *Nat. Commun.* **12**, 2588 (2021).

57. W. Liu, Y. Zhang, F. L. Tissot, G. Avice, Z. Ye, Q. Z. Yin, I/Pu reveals Earth mainly accreted from volatile-poor differentiated planetesimals. *Sci. Adv.* **9**, eadg9213 (2023).

58. W. Wang, M. J. Walter, J. P. Brodholt, S. Huang, Early planetesimal differentiation and late accretion shaped Earth's nitrogen budget. *Nat. Commun.* **15**, 4169 (2024).

59. Z. Wang, H. Becker, Chalcophile elements in Martian meteorites indicate low sulfur content in the Martian interior and a volatile element-depleted late veneer. *Earth Planet. Sci. Lett.* **463**, 56–68 (2017).

60. F. Nimmo, T. Kleine, A. Morbidelli, D. Nesvorny, Mechanisms and timing of carbonaceous chondrite delivery to the Earth. *Earth Planet. Sci. Lett.* **648**, 119112 (2024).

61. G. Fiquet, A. L. Auzende, J. Siebert, A. Corgne, H. Bureau, H. Ozawa, G. Garbarino, Melting of peridotite to 140 gigapascals. *Science* **329**, 1516–1518 (2010).

62. D. Andrault, N. Bolfan-Casanova, G. L. Nigro, M. A. Bouhifd, G. Garbarino, M. Mezouar, Solidus and liquidus profiles of chondritic mantle: Implication for melting of the Earth across its history. *Earth Planet. Sci. Lett.* **304**, 251–259 (2011).

63. M. J. Walter, Y. Thibault, Partitioning of tungsten and molybdenum between metallic liquid and silicate melt. *Science* **270**, 1186–1189 (1995).

64. Y. Akahama, H. Kawamura, High-pressure Raman spectroscopy of diamond anvils to 250 GPa: Method for pressure determination in the multimegabar pressure range. *J. Appl. Phys.* **96**, 3748–3751 (2004).

65. A. Boujibar, D. Andrault, M. A. Bouhifd, N. Bolfan-Casanova, J. L. Devidal, N. Trcera, Metal-silicate partitioning of sulphur, new experimental and thermodynamic constraints on planetary accretion. *Earth Planet. Sci. Lett.* **391**, 42–54 (2014).

66. B. J. Wood, E. S. Kiseeva, F. J. Mirolo, Accretion and core formation: The effects of sulfur on metal-silicate partition coefficients. *Geochim. Cosmochim. Acta* **145**, 248–267 (2014).

67. D. Huang, J. Siebert, J. Badro, High pressure partitioning behavior of Mo and W and late sulfur delivery during Earth's core formation. *Geochim. Cosmochim. Acta* **310**, 19–31 (2021).

68. E. S. Jennings, S. A. Jacobson, D. C. Rubie, Y. Nakajima, A. Vogel, L. A. Rose-Weston, D. J. Frost, Metal-silicate partitioning of W and Mo and the role of carbon in controlling their abundances in the bulk silicate Earth. *Geochim. Cosmochim. Acta* **293**, 40–69 (2021).

69. E. S. Jennings, J. Wade, V. Laurenz, S. Petitgirard, Diamond anvil cell partitioning experiments for accretion and core formation: Testing the limitations of electron microprobe analysis. *Microsc. Microanal.* **25**, 1–10 (2019).

70. G. Martínez-Criado, J. Villanova, R. Tucoulou, D. Salomon, J. P. Suuronen, S. Labouré, C. Guilloud, V. Valls, R. Barrett, E. Gagliardini, Y. Dabin, R. Baker, S. Bohic, C. Cohen, J. Morse, ID16B: A hard x-ray nanoprobe beamline at the ESRF for nano-analysis. *J. Synchrotron Radiat.* **23**, 344–352 (2016).

71. I. Blanchard, S. Petitgirard, V. Laurenz, N. Miyajima, M. Wilke, D. C. Rubie, S. S. Lobanov, L. Hennet, W. Morgenroth, R. Tucoulou, V. Bonino, X. Zhao, I. Franchi, Chemical analysis of trace elements at the nanoscale in samples recovered from laser-heated diamond anvil cell experiments. *Phys. Chem. Miner.* **49**, 18 (2022b).

72. V. A. Solé, E. Papillon, M. Cotte, P. Walter, J. Susini, A multiplatform code for the analysis of energy-dispersive x-ray fluorescence spectra. *Spectrochim. Acta B Spectrosc.* **62**, 63–68 (2007).

73. T. Vincent, V. Valls, payno, J. Kieffer, V. A. Solé, P. Paleo, dnaudet, W. De Nolf, Pierre K., J. Garriga, M. Retegan, M. Rovezzi, H. Fangohr, P. Kenter, T. Fuchs, L. Huder, UUSim, V.

Favre-Nicolin, CaptainNemoz, C. Kang, picca, S. W. Kim, T. A. Caswell, J. P. C. Bertoldo, R. N. Watanabe, L. Pithan, lesaintjerome, carez, T. Farago, schooft, silx-kit/silx: 2.1.0: 2024/04/19, version 2.1.0. Zenodo (2024); https://doi.org/10.5281/zenodo.10996641.

74. A. M. Davis, F. M. Richter, "Condensation and evaporation of Solar System materials" in *Treatise on Geochemistry*, H. D. Holland, K. K. Turekian, Eds. (Elsevier, ed. 2, 2003), p. 711.

75. C. A. Norris, B. J. Wood, Earth's volatile contents established by melting and vaporization. *Nature* **549**, 507–510 (2017).

76. D. Huang, J. Siebert, P. Sossi, E. Kubik, G. Avice, M. Murakami, Nitrogen sequestration in the core at megabar pressure and implications for terrestrial accretion. *Geochim. Cosmochim. Acta* **376**, 100–112 (2024).

77. E. Cottrell, D. Walker, Constraints on core formation from Pt partitioning in mafic silicate liquids at high temperatures. *Geochim. Cosmochim. Acta* **70**, 1565–1580 (2006).

78. J. Wade, B. J. Wood, J. Tuff, Metal-silicate partitioning of Mo and W at high pressures and temperatures: Evidence for late accretion of sulphur to the Earth. *Geochim. Cosmochim. Acta* **85**, 58–74 (2012).

79. I. Blanchard, J. Siebert, S. Borensztajn, J. Badro, The solubility of heat-producing elements in Earth's core. *Geochem. Perspect. Lett.* **5**, 1–5 (2017).

80. I. Blanchard, S. Abeykoon, D. J. Frost, D. C. Rubie, Sulfur content at sulfide saturation of peridotitic melt at upper mantle conditions. *Am. Min.* **106**, 1835–1843 (2021).

81. D. J. Lindstrom, J. H. Jones, Neutron activation analysis of multiple 10–100 µg glass samples from siderophile element partitioning experiments. *Geochim. Cosmochim. Acta* **60**, 1195–1203 (1996).

82. A. Borisov, H. Palme, Experimental determination of the solubility of platinum in silicate melts. *Geochim. Cosmochim. Acta* **61**, 4349–4357 (1997).

83. A. Holzheid, P. Sylvester, H. S. C. O'Neill, D. C. Rubie, H. Palme, Evidence for a late chondritic veneer in the Earth's mantle from high-pressure partitioning of palladium and platinum. *Nature* **406**, 396–399 (2000).

84. W. Ertel, M. J. Walter, M. J. Drake, P. J. Sylvester, Experimental study of platinum solubility in silicate melt to 14 GPa and 2273 K: Implications for accretion and core formation in Earth. *Geochim. Cosmochim. Acta* **70**, 2591–2602 (2006).

85. A. Holzheid, T. L. Grove, Sulfur saturation limits in silicate melts and their implications for core formation scenarios for terrestrial planets. *Am. Min.* **87**, 227–237 (2002).

86. J. Li, C. Agee, Element partitioning constraints on the light element composition of the Earth's core. *Geophys. Res. Lett.* **28**, 81–84 (2001).

87. C. Fincham, F. D. Richardson, The behaviour of sulphur in silicate and aluminate melts. *Proc. R. Soc. Lond. A.* **223**, 40–62 (1954).

88. J. A. Mavrogenes, H. S. C. O'Neill, The relative effects of pressure, temperature and oxygen fugacity on the solubility of sulfide in mafic magmas. *Geochim. Cosmochim. Acta* **63**, 1173–1180 (1999).

89. D. R. Haughton, P. L. Roeder, B. J. Skinner, Solubility of sulfur in mafic magmas. *Econ. Geol.* **69**, 451–467 (1974).

90. K. Tsuno, E. Ohtani, H. Terasaki, Immiscible two-liquid regions in the Fe-O-S system at high pressure: Implications for planetary cores. *Phys. Earth Planet. Inter.* **160**, 75–85 (2007).

91. G. Morard, T. Katsura, Pressure-temperature cartography of Fe-S-Si immiscible system. *Geochim. Cosmochim. Acta* **74**, 3659–3667 (2010).

92. J. Siebert, V. Malavergne, F. Guyot, R. Combes, I. Martinez, The behaviour of sulphur in metal-silicate core segregation experiments under reducing conditions. *Phys. Earth Planet. Inter.* **143**, 433–443 (2004).

93. Z. Ma, Thermodynamic description for concentrated metallic solutions using interaction parameters. *Metall. Mater. Trans. B* **32**, 87–103 (2001).

94. M. Suzumebe, *Japan Society for the Promotion of Science and Nineteenth Committee on Steelmaking, Steelmaking Data Sourcebook* (Gordon and Breach Science Publishers, 1988).

95. N. L. Chabot, C. B. Agee, Core formation in the earth and moon: New experimental constraints from V, Cr, and Mn. *Geochim. Cosmochim. Acta* **67**, 2077–2091 (2003).

96. B. J. Wood, S. G. Nielsen, M. Rehkämper, A. N. Halliday, The effects of core formation on the Pb- and Tl- isotopic composition of the silicate Earth. *Earth Planet. Sci. Lett.* **269**, 326–336 (2008).

97. K. Righter, M. Humayun, A. J. Campbell, L. Danielson, D. Hill, M. J. Drake, Experimental studies of metal-silicate partitioning of Sb: Implications for the terrestrial and lunar mantles. *Geochim. Cosmochim. Acta* **73**, 1487–1504 (2009).

98. J. Siebert, A. Corgne, F. J. Ryerson, Systematics of metal-silicate partitioning for many siderophile elements applied to Earth's core formation. *Geochim. Cosmochim. Acta* **75**, 1451–1489 (2011).

99. C. Ballhaus, R. O. C. Fonseca, C. Münker, A. Rohrbach, T. J. Nagel, I. M. Speelmanns, H. M. Helmy, A. Zirner, A. K. Vogel, A. Heuser, The great sulfur depletion of Earth's mantle is not a signature of mantle-core equilibration. *Contrib. Mineral. Petrol.* **172**, 1–10 (2017).

100. K. Righter, K. Pando, N. Marin, D. Ross, M. Righter, L. Danielson, T. Lapen, C. Lee, Volatile element signatures in the mantles of Earth, Moon, and Mars: Core formation fingerprints from Bi, Cd, In, and Sn. *Meteorit. Planet. Sci.* **53**, 284–305 (2018).

101. P. O'Brien, K. J. Walsh, A. Morbidelli, S. N. Raymond, A. M. Mandell, Water delivery and giant impacts in the 'Grand Tack' scenario. *Icarus* **239**, 74–84 (2014).

102. S. B. Jacobsen, The Hf-W isotopic system and the origin of the Earth and Moon. *Annu. Rev. Earth Planet. Sci.* **33**, 531–570 (2005).

103. B. J. Wood, M. J. Walter, J. Wade, Accretion of the Earth and segregation of its core. *Nature* **441**, 825–833 (2006).

104. A. N. Halliday, B. J. Wood, Geophysics. How did Earth accrete? *Science* **325**, 44–45 (2009).

105. J. F. Rudge, T. Kleine, B. Bourdon, Broad bounds on Earth's accretion and core formation constrained by geochemical models. *Nat. Geosci.* **3**, 439–443 (2010).

106. W. K. Hartmann, D. R. Davis, Satellite-sized planetesimals and lunar origin. *Icarus* **24**, 504 (1975).

107. A. G. W. Cameron, W. Benz, The origin of the Moon and the single impact hypothesis IV. *Icarus* **92**, 204–216 (1991).

108. R. M. Canup, Simulations of a late lunar-forming impact. *Icarus* **168**, 433–456 (2004).

109. R. Deguen, M. Landeau, P. Olson, Turbulent metal-silicate mixing, fragmentation, and equilibration in magma oceans. *Earth Planet. Sci. Lett.* **391**, 274–287 (2014).

110. J. Badro, J. P. Brodholt, H. Piet, J. Siebert, F. J. Ryerson, Core formation and core composition from coupled geochemical and geophysical constraints. *Proc. Natl. Acad. Sci. U.S.A.* **112**, 12310–12314 (2015).

111. J. Siebert, J. Badro, D. Antonangeli, F. J. Ryerson, Terrestrial accretion under oxidizing conditions. *Science* **339**, 1194–1197 (2013).

112. A. N. Halliday, Mixing, volatile loss and compositional change during impact-driven accretion of the Earth. *Nature* **427**, 505–509 (2004).

113. T. Kleine, K. Mezger, H. Palme, C. Münker, The W isotope evolution of the bulk silicate Earth: Constraints on the timing and mechanisms of core formation and accretion. *Earth Planet. Sci. Lett.* **228**, 109–123 (2004).

114. Y. Li, R. Dasgupta, K. Tsuno, The effects of sulfur, silicon, water, and oxygen fugacity on carbon solubility and partitioning in Fe-rich alloy and silicate melt systems at 3 GPa and

1600°C: Implications for core-mantle differentiation and degassing of magma oceans and reduced planetary mantles. *Earth Planet. Sci. Lett.* **415**, 54–66 (2015).

115. F. Gaillard, F. Bernadou, M. Roskosz, M. A. Bouhifd, Y. Marrocchi, G. Iacono-Marziano, M. Moreira, B. Scaillet, G. Rogerie, Redox controls during magma ocean degassing. *Earth Planet. Sci. Lett.* **577**, 117255 (2022).

116. J. T. Gu, B. Peng, X. Ji, J. Zhang, H. Yang, S. Hoyos, M. M. Hirschmann, E. S. Kite, R. A. Fischer, Composition of Earth's initial atmosphere and fate of accreted volatiles set by core formation and magma ocean redox evolution. *Earth Planet. Sci. Lett.* **629**, 118618 (2024).

117. V. Laurenz, D. C. Rubie, D. J. Frost, A. K. Vogel, The importance of sulfur for the behavior of highly-siderophile elements during Earth's differentiation. *Geochim. Cosmochim. Acta* **194**, 123–138 (2016).

118. D. C. Rubie, V. Laurenz, S. A. Jacobson, A. Morbidelli, H. Palme, A. K. Vogel, D. J. Frost, Highly siderophile elements were stripped from Earth's mantle by iron sulfide segregation. *Science* **353**, 1141–1144 (2016).

119. E. S. Steenstra, O. T. Lord, S. Vitale, E. S. Bullock, S. Klemme, M. Walter, Sulfur solubility in a deep magma ocean and implications for the deep sulfur cycle. *Geochem. Perspect. Lett.* **22**, 5–9 (2022).

120. I. Blanchard, J. Siebert, E. Kubik, A. Minchenkova, L. Calvo, N. Wehr, Earth's deep magma ocean never reached sulfide saturation. *Geochem. Perspect. Lett.* **34**, 6–10 (2025).

121. J. L. Wykes, H. S. C. O'Neill, J. A. Mavrogenes, The effect of FeO on the sulfur content at sulfide saturation (SCSS) and the selenium content at selenide saturation of silicate melts. *J. Petrol.* **56**, 1407–1424 (2015).

122. R. Caracas, K. Hirose, R. Nomura, M. D. Ballmer, Melt-crystal density crossover in a deep magma ocean. *Earth Planet. Sci. Lett.* **516**, 202–211 (2019).

123. K. Armstrong, D. J. Frost, C. A. McCammon, D. C. Rubie, T. B. Ballaran, Deep magma ocean formation set the oxidation state of Earth's mantle. *Science* **365**, 903–906 (2019).

124. H. L. Zhang, M. M. Hirschmann, O. T. Lord, A. Rosenthal, S. Yaroslavtsev, E. Cottrell, A. I. Chumakov, M. J. Walter, Ferric iron stabilization at deep magma ocean conditions. *Sci. Adv.* **10**, eadp1752 (2024).

125. S. C. O'Neill, S. M. Eggins, The effect of melt composition on trace element partitioning: An experimental investigation of the activity coefficients of FeO, NiO, CoO, MoO_2 and MoO_3 in silicate melts. *Chem. Geol.* **186**, 151–181 (2002).

126. G. Dreibus, H. Palme, B. Spettel, J. Zipfel, H. Wänke, Sulfur and selenium in chondritic meteorites. *Meteoritics* **30**, 439–445 (1995).

127. P. G. Brown, A. R. Hildebrand, M. E. Zolensky, M. Grady, R. N. Clayton, T. K. Mayeda, E. Tagliaferri, R. Spalding, N. D. MacRae, E. L. Hoffman, D. W. Mittlefehldt, J. F. Wacker, J. A. Bird, M. D. Campbell, R. Carpenter, H. Gingerich, M. Glatiotis, E. Greiner, M. J. Mazur, P. J. A. McCausland, H. Plotkin, T. R. Mazur, The fall, recovery, orbit, and composition of the Tagish Lake meteorite: A new type of carbonaceous chondrite. *Science* **290**, 320–325 (2000).

128. J. M. Friedrich, M.-S. Wang, M. E. Lipschutz, Comparison of the trace element composition of Tagish Lake with other primitive carbonaceous chondrites. *Meteorit. Planet. Sci.* **37**, 677–686 (2002).

129. S. F. Wolf, D. L. Unger, J. M. Friedrich, Determination of cosmochemically volatile trace elements in chondritic meteorites by inductively coupled plasma mass spectrometry. *Anal. Chim. Acta* **528**, 121–128 (2005).

130. Z. Wang, H. Becker, T. Gawronski, Partial re-equilibration of highly siderophile elements and the chalcogens in the mantle: A case study on the Baldissero and Balmuccia peridotite massifs (Ivrea Zone, Italian Alps). *Geochim. Cosmochim. Acta* **108**, 21–44 (2013).

131. J. L. Hellmann, T. Hopp, C. Burkhardt, H. Becker, M. Fischer-Gödde, T. Kleine, Tellurium isotope cosmochemistry: Implications for volatile fractionation in chondrite parent bodies and origin of the late veneer. *Geochim. Cosmochim. Acta* **309**, 313–328 (2021).
